# A passage-dependent network for estimating the *in vitro* senescence of mesenchymal stromal/stem cells using microarray, bulk and single cell RNA sequencing

**DOI:** 10.3389/fcell.2023.998666

**Published:** 2023-02-07

**Authors:** Yong Yang, Wencheng Zhang, Xicheng Wang, Jingxian Yang, Yangyang Cui, Haimeng Song, Weiping Li, Wei Li, Le Wu, Yao Du, Zhiying He, Jun Shi, Jiangnan Zhang

**Affiliations:** ^1^ Department of General Surgery, The First Affiliated Hospital of Nanchang University, Nanchang, Jiangxi, China; ^2^ Institute for Regenerative Medicine, Shanghai East Hospital, School of Life Sciences and Technology, Tongji University, Shanghai, China; ^3^ Shanghai Engineering Research Center of Stem Cells Translational Medicine, Shanghai, China; ^4^ Shanghai Institute of Stem Cell Research and Clinical Translation, Shanghai, China; ^5^ Department of Anesthesiology, The First Affiliated Hospital of Nanchang University, Nanchang, Jiangxi, China; ^6^ Postgraduate Training Base of Shanghai East Hospital, Jinzhou Medical University, Jinzhou, Liaoning, China; ^7^ Department of Gastrointestinal Surgery, The First People’s Hospital of Taicang City, Taicang Affiliated Hospital of Soochow University, Taicang, Jiangsu, China; ^8^ Department of General Surgery, Fuzhou Dongxiang District People’s Hospital, Fuzhou, Jiangxi, China

**Keywords:** mesenchymal stem cells (MSCs), replicative senescence, extracellular matrix (ECM), microRNAs (miRNAs), *in vitro* expansion, regulatory network construction

## Abstract

Long-term *in vitro* culture of human mesenchymal stem cells (MSCs) leads to cell lifespan shortening and growth stagnation due to cell senescence. Here, using sequencing data generated in the public domain, we have established a specific regulatory network of “transcription factor (TF)-microRNA (miRNA)-Target” to provide key molecules for evaluating the passage-dependent replicative senescence of mesenchymal stem cells for the quality control and status evaluation of mesenchymal stem cells prepared by different procedures. Short time-series expression miner (STEM) analysis was performed on the RNA-seq and miRNA-seq databases of mesenchymal stem cells from various passages to reveal the dynamic passage-related changes of miRNAs and mRNAs. Potential miRNA targets were predicted using seven miRNA target prediction databases, including TargetScan, miRTarBase, miRDB, miRWalk, RNA22, RNAinter, and TargetMiner. Then use the TransmiR v2.0 database to obtain experimental-supported transcription factor for regulating the selected miRNA. More than ten sequencing data related to mesenchymal stem cells or mesenchymal stem cells reprogramming were used to validate key miRNAs and mRNAs. And gene set variation analysis (GSVA) was performed to calculate the passage-dependent signature. The results showed that during the passage of mesenchymal stem cells, a total of 29 miRNAs were gradually downregulated and 210 mRNA were gradually upregulated. Enrichment analysis showed that the 29 miRNAs acted as multipotent regulatory factors of stem cells and participated in a variety of signaling pathways, including TGF-beta, HIPPO and oxygen related pathways. 210 mRNAs were involved in cell senescence. According to the target prediction results, the targets of these key miRNAs and mRNAs intersect to form a regulatory network of “TF-miRNA-Target” related to replicative senescence of cultured mesenchymal stem cells, across 35 transcription factor, 7 miRNAs (has-mir-454-3p, has-mir-196b-5p, has-mir-130b-5p, has-mir-1271-5p, has-let-7i-5p, has-let-7a-5p, and has-let-7b-5p) and 7 predicted targets (PRUNE2, DIO2, CPA4, PRKAA2, DMD, DDAH1, and GATA6). This network was further validated by analyzing datasets from a variety of mesenchymal stem cells subculture and lineage reprogramming studies, as well as qPCR analysis of early passages mesenchymal stem cells versus mesenchymal stem cells with senescence morphologies (SA-**β**-Gal^+^). The “TF-miRNA-Target” regulatory network constructed in this study reveals the functional mechanism of miRNAs in promoting the senescence of MSCs during *in vitro* expansion and provides indicators for monitoring the quality of functional mesenchymal stem cells during the preparation and clinical application.

## Introduction

Mesenchymal stem cells (MSCs) are multipotent stromal cells with differentiation potentials, paracrine and immunomodulatory properties. They can be isolated from multiple sources such as bone marrow, perinatal tissue or adipose tissue, and expanded *in vitro* through standardized procedures, making them the most popular and ideal candidate cell types for tissue regeneration or disease treatment. The quality of MSC product is known to rely on the isolation and expansion procedure other than the source and genetic background of the donor tissues. Therefore, the development of *in vitro* expansion protocol is crucial to promote the therapeutic application of MSCs. However, at present, different researchers and manufacturers have adopted a wide range of protocols to prepare MSC based products (Mendicino et al., 2014). Previous studies have shown that the expression levels of CD105, CD106, and CD146 were significantly decreased after prolonged *in vitro* culture and repeated passage of MSCs (Yang et al., 2018). And it has been reported that the surface expression of CD105 may be affected by the culture protocols (Das et al., 2019).

Although the International Society for Cellular Therapy (ISCT) has proposed a set of markers and feature to characterize MSCs (Dominici et al., 2006), those markers aren’t sufficient for the quality control along the expansion of the MSC-product (Lukomska et al., 2019). Of all the factors that have been confirmed to affect the proliferation of stem cells to date, cell senescence is an inevitable important factor, usually accompanied by a decrease in cell replication ability and functional changes (Xiao et al., 2021). Therefore, understanding the mechanism of *in vitro* senescence of MSCs might provide novel insights for the rejuvenation of MSCs.

Senescence is often described as cell cycle arrest, accumulation of which participates in aging and aging related diseases mainly through their secretory activity, which is commonly referred to as senescence related secretory phenotype SASP. The DNA damage, nuclear structure change and chromatin rearrangement are involved in the signal pathways leading to changes in the transcription and secretion of SASP component genes. The regulatory mechanism of cell senescence is complicated. However, recently, increasing evidence shows that miRNAs play a key role in regulating cell senescence MSCs (Hong et al., 2020).

miRNAs are a class of non-coding RNAs about 21–23 nucleotides long. They become key inhibitors of gene expression by binding to the 3′- untranslated region (UTR) of target mRNAs, and participate in the proliferation, differentiation and survival of stem cells (Xu et al., 2018). In MSCs, miR-155-5p was discovered to regulate cell senescence by inhibiting mitochondrial fission and increasing mitochondrial fusion in MSCs through AMPK signaling pathway. Therefore, inhibition of miR-155-5p can restore MSC vitality to improve the cardiac protection after infarction (Hong et al., 2020). MiR-146a and miR-29b-3p expressed in the MSCs derived exosomes were also found to be key factors in mediating oxidative stress induced cell senescence in other cell types (such as endothelial cells) (Su et al., 2019; Xiao et al., 2021). These regulatory functions of miRNAs are usually achieved by targeting a broad spectrum of genes involved in one or more signaling pathways.

Previously, by comparing the genetic signature of four stages of hepatic lineages, including human biliary tree stem cells (BTSCs), human hepatic stem cells (hHPSCs), human hepatoblasts (hHBs) and adult hepatocytes (AHEPs), we developed a “TF-miRNA-target” network, which can be used to determine the hepatic differentiation or maturation of stem cells or progenitors (Wang X. et al., 2021) (Yang et al., 2018). Using a large amount of sequencing data generated in the public domain by different groups conducting basic, transformation and clinical research or application of MSCs, a general model can be built for quality control and status evaluation of MSCs prepared by different procedures.

In this study, we used the sequencing data, miRNA-seq and bulk RNA-seq data to elucidate the regulatory characteristics of miRNA and mRNA expression from early to late passage of MSCs. Based on the *in vitro* senescence dataset of MSCs and the sequencing data related to *in vivo* senescence and reprogramming, we have constructed a MSC-related “TF-miRNA-target” network for evaluating the quality of MSCs which provides a feasible scheme for large-scale expansion of MSCs in the future.

## Methods and materials

### Data source

All 12 datasets were collected from the Gene Expression Omnius (GEO) (http://www.ncbi.nlm.nih.gov/GEO) (Table 1). All data were standardized with the pheatmap R software package and displayed on the heat map. In addition, the heatmap had been scaled to better show differences and express changes. See Table 1 for details of these data.

**TABLE 1:** The detailed information of 12 datasets analyzed in the present study.

Dataset	Species	Platform	Data type	Team/References
GSE178514	*Homo sapiens*	GPL23227 BGISEQ-500	Bulk RNA-seq	Wang S. et al. (2021)
GSE139073	*Homo sapiens*	GPL18460	Bulk RNA-seq	Andrzejewska et al. (2019)
		Illumina HiSeq 1,500		
		GPL20301		
		Illumina HiSeq 4,000		
GSE146247	*Homo sapiens*	GPL24676	Bulk RNA-seq	Bi et al. (2020)
		Illumina NovaSeq 6,000		
GSE120800	*Homo sapiens*	GPL16791	Bulk RNA-seq	Jiao et al. (2021)
		Illumina HiSeq 2,500		
GSE115068	*Mus musculus*	GPL21163	Microarray	Taketani et al. (2019)
		Agilent-074809 SurePrint G3 Mouse GE v2 8 × 60 K Microarray		
GSE145477	*Mus musculus*	GPL21493	scRNA-seq	Zhong et al. (2020)
		Illumina HiSeq 3,000		Zhong et al. (2022)
GSE25069	*Mus musculus*	GPL10192	Microarray	Nodari et al. (2021)
		NimbleGen *Mus musculus* MM9 Expression Array		
GSE183995	*Homo sapiens*	GPL16686	Microarray	Kim et al. (2021)
		[HuGene-2_0-st] Affymetrix Human Gene		
		2.0 ST Array		
GSE137186	*Homo sapiens*	GPL20301	Bulk RNA-seq	Salerno et al. (2020)
		Illumina HiSeq 4,000		
GSE110755	*Homo sapiens*	GPL16699	Microarray	Lam et al. (2018)
		Agilent-039494 SurePrint G3 Human GE v2 8 × 60 K Microarray		
GSE39035	*Homo sapiens*	GPL13607	Microarray	Kilpinen et al. (2013)
		Agilent-028004 SurePrint G3 Human GE 8 × 60 K Microarray		
GSE117837	*Homo sapiens*	GPL16791	scRNA-seq	Huang et al. (2019)
		Illumina HiSeq 2,500		

### Cell sources

MSCs were obtained from the discarded umbilical cord tissues and prepared and stored in the GMP laboratory of Shanghai East Hospital. The use of the human samples and cells has received approval from the Ethics Committee of Shanghai East Hospital. P1 UC-MSCs were provided free of charge by GMP facilities for validation experiments. Cells were expanded under 2D attachment condition with a serum free medium (StemPro® MSC SFM, Thermofisher, MA, United States) for up to 15 passages at a ratio of 1:3 every 4 days. Cells at passages P4 were used for the characterization of the ISCT-recommended cell surface characteristics of MSCs, including expression of CD73, CD90, and CD105 (>95%) and no presentation of CD34, CD45, CD14 or CD11b, CD79α or CD19, and HLA-DR.

### STEM analysis

The bulk RNA-seq and miRNA-seq data of MSCs of different passages were collected to conduct STEM analysis using STEM v1.3.13 software (Ernst and Bar-Joseph 2006). All mRNAs and miRNAs from GSE178514 were layered into different profiles according to various expression patterns calculated by STEM analysis, respectively. Three passages of MSCs, including P4, P6, and P12, were considered to be at different time points.

### Gene ontology (GO) and kyoto encyclopedia of genes and genome (KEGG) enrichment analysis

In order to explore the biological function of a spectrum of genes specific to the passage of MSCs, KEGG, and GO enrichment analyses were performed using clusterProfiler R package (Yu et al., 2012). In addition, this study used the DIANA miRPath v3.0 database, which can quickly predict the potential targets of miRNAs, and then efficiently run KEGG path analysis to study the possible functions of miRNAs (Vlachos et al., 2015). Only the top significant terms were showed in figures.

### Target prediction databases of miRNAs

In the current study, we used seven databases to predict the targets of 29 miRNAs, including TargetScan (http://www.targetscan.org/vert_72/) (Lewis et al., 2003), miRTarBase (http://mirtarbase.mbc.nctu.edu.tw/php/index.php) (Chou et al., 2018), miRDB (http://www.mirdb.org/) (Chen and Wang 2020), miRWalk (http://mirwalk.umm.uni-heidelberg.de/) (Sticht et al., 2018), RNA22 (https://cm.jefferson.edu/rna22/) (Miranda et al., 2006), RNAinter (http://www.rnainter.org/) (Kang et al., 2022), TargetMiner (https://www.isical.ac.in/) (Bandyopadhyay and Mitra 2009). “*p* < 0.05” was considered statistical significance.

### Principal component analysis and three-dimensional principal component analysis (PCA)

The RNA sequence and miRNA sequence data of cultured cells were analyzed by PCA or 3D PCA to check the performance of seven miRNAs and seven gene signatures. The scRNA-seq data about *in vitro* passaged and stimulated MSCs were collected and performed single cell RNA-seq analysis using Seurat (version: 4.1.1) (Hao et al., 2021) to verify the characteristics of seven genes and their correlation with oxygen related pathways.

### Constructing a “TF-miRNA-target” regulatory network for *in vitro* cultured MSCs

Experimentally-validated interactions between passage-related miRNAs and their regulatory TFs were retrieved from the TransmiR v2.0 database (Tong et al., 2019). The “TF-miRNA-target” regulatory network related to MSCs passage was constructed using Cytocape Java Version 3.7.1 (https://cytoscape.org) software (Tong, et al., 2019).

### Correlation analysis

The correlation coefficients between the miRNA and the target or between the seven-gene signature and the interested signaling pathways was calculated. *p* < 0.05 was statistically significant. The specific correlation coefficient is shown in figures.

### Gene set variation analysis (GSVA) and single sample gene set enrichment analysis (ssGSEA)

The RNA-seq data associated with MSCs subculture and single cell sequencing data were scored using GSVA/ssGSEA algorithm, with each sample/cell obtaining a GSVA score (Hanzelmann et al., 2013).

### SA-**β**-gal senescence assay

Cultured MSCs of early passage (P3) and late passage (P10, P12) were selected for the senescence assay following the instructions of the SA- **β**-gal senescence assay kit (Beyotime, Shanghai, China). Briefly, cells were washed with 1 × PBS for three times, 5 min each time. Then, add an appropriate amount of fixation buffer and incubate at room temperature for 15 min. After three times of washing, 5 min each time, cells were incubated overnight at 37°C with fresh SA-**β**-gal solution for senescence assay. Experiments were run with three preps of cells for both the early passages and the late passages MSCs.

### qRT-PCR

Total RNA was extracted from MSCs of early passage (P3) and late passage (P10 and P12) using Trizol (Thermofisher, MA, United States). First-strand cDNA was synthesized using the Primescript first strand cDNA synthesis kit (Takara Bio Inc., Shiga, Japan) was used as a template for PCR amplification. Primers for the 7 genes were obtained from the primerbank (https://pga.mgh.harvard.edu/primerbank/) and synthesized by Sangon Biotech (Shanghai, China). Mixtures were annealed at 50°C for 2 min and 95°C for 10 min, followed by 40 cycles of 95°C (15 s) and 60°C (1 min). Expression of glyceraldehyde-3-phosphate dehydrogenase (GAPDH) was used as a control and a standard. Primers are listed in Supplementary Table S1.

### Statistical analysis

Statistically significant differences were calculated using statistical methods including T-test and Wilcoxon test. *p*-values less than 0.05, 0.01, 0.001, and 0.0001 were considered statistically significant, and asterisks were used to show differentially expressed results, shown as “*”, “**”, “***”, and “****”, respectively.

## Result

### 29 miRNAs were gradually downregulated during the *in vitro* passage of MSCs

**FIGURE 1:**
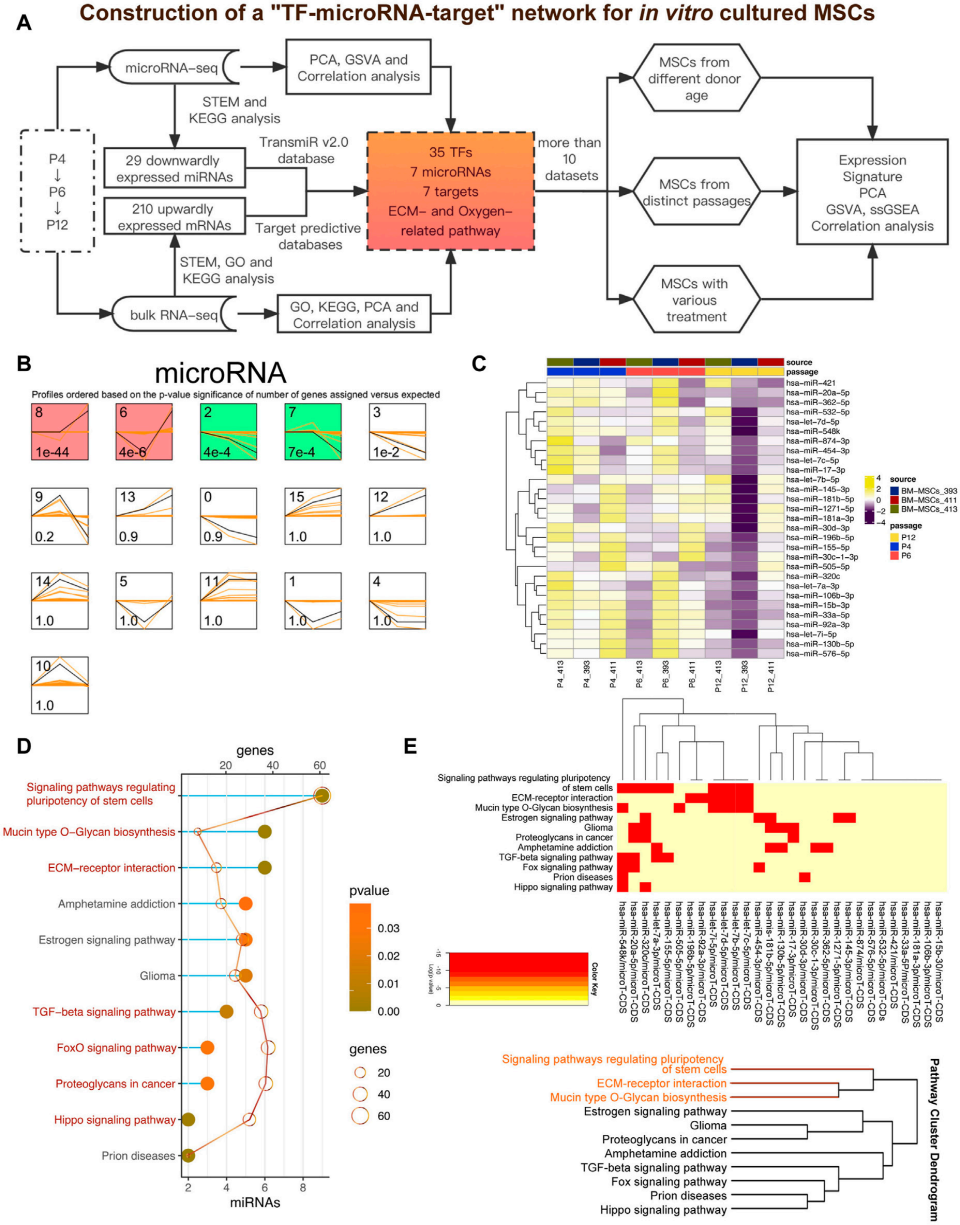
Downwardly expressed miRNAs during the passage of MSCs *in vitro*. **(A)** Pipeline of the present study. GSE178514 dataset was used for identifying and constructing the network while other datasets were adopted to validate the results. **(B)** STEM analysis of miRNA-seq data. Significant profiles are colored, and the same color represents a similar expression pattern. Significant miRNA profiles are colored, and the same color represents a similar expression pattern, such as gradually increasing or decreasing pattern. **(C)** Heatmap plots 29 gradually downregulated miRNAs, which was obtained from miRNA Profile 2. **(D)** KEGG analysis of 29 miRNAs *via* the DIANA-miRPath database. Top significant pathways were presented. **(E)** Clustering analysis of the enriched pathways and the involved miRNAs.

*In vitro* expansion of MSCs played a crucial role in culturing enough MSCs products for clinical use. In the present study, we tried to explore the potential relationship between miRNA and mRNA profiles to provide insights into the dynamic changes of MSCs during the *in vitro* expansion (Figure 1A). We collected miRNA-seq and RNA-seq data of MSCs of passage 4 (P4), passage 6 (P6) and passage 12 (P12) from GSE178514 (Wang S. et al., 2021), and ran STEM to study the changing genes in MSCs in the late passage compared with the early passage (Ernst and Bar-Joseph 2006). In Figure 1C, all miRNAs were clustered into 16 profiles but only four profiles were considered as significant (colored profile), including Profile 8, Profile 6, Profile 2 and Profile 7 (Figure 1B). Among the four profiles, Profile 2 presented a gradually downregulated trend, indicating its potential function associated with senescence during *in vitro* expansion of MSCs (Figure 1C). Therefore, we further studied the potential functions of 29 miRNAs in Profile 2 by using the network serve web-server DIANA-miRPath v3.0 databases for a miRNA pathway analysis (https://dianalab.e-ce.uth.gr/html/mirpathv3/index.php?r=mirpath) (Vlachos, et al., 2015). As shown in Figure 1D, most of the enrichment pathways of 29 miRNAs were related to MSC stemness, including “Signaling pathways regulating pluripotency of stem cells” (Supplementary Figure S1), “TGF−**β** signaling pathway,” “Hippo signaling pathway,” and “FoxO signaling pathway”. It is worth noting that several terms related to extracellular matrix (ECM) had also been enriched, involving “Mucin type O−Glycan biosynthesis,” “ECM−receptor interaction” (Supplementary Figure S2) and “Proteoglycans in cancer.” Of note, using clustering analysis, we found that ECM and stemness were clustered together, implying that the MSCs stemness might be closely pertinent with the deposition or balances of ECM (Figure 1E).

### 210 genes were correlated with the *in vitro* senescence of MSCs

**FIGURE 2:**
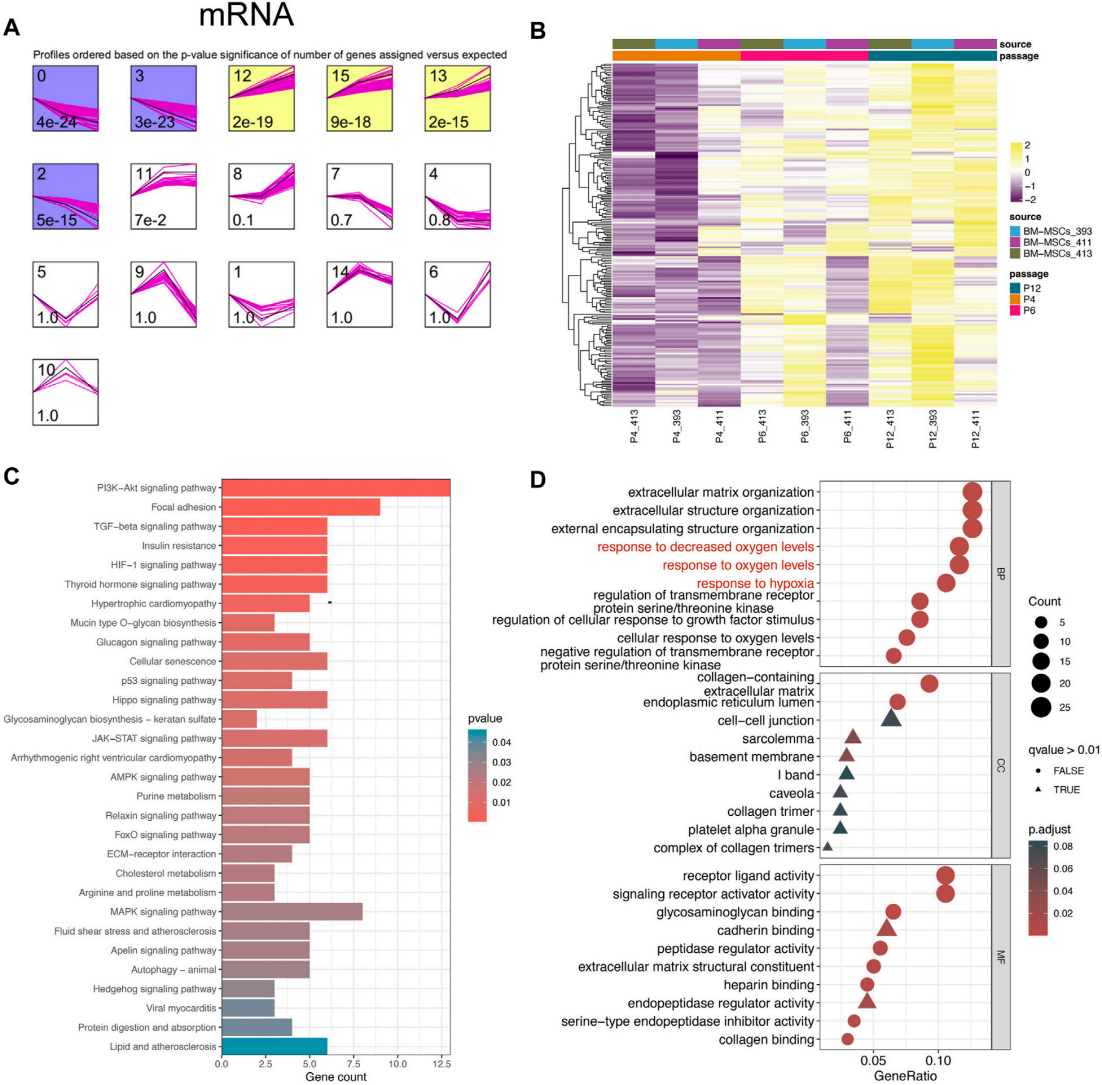
Upwardly expressed mRNAs in MSCs cultured *in vitro*. **(A)** STEM analysis of bulk RNA-seq data. **(B)** Heatmap plots 210 mRNAs that were gradually upregulated from P4 to p12. **(C)** KEGG analysis of 210 genes. The top 30 important terms are displayed. **(D)** GO analysis of 210 genes. The top 10 crucial terms for biological process (BP), cellular component (CC), and molecular function (MF) are presented.

In view of the downregulation of 29 miRNAs after MSC passage and their high correlation with MSC stem cells, this led us to explore whether there are also some mRNA with specific dynamic changes during MSC passage, especially those genes that may be upregulated by miRNAs (Chivukula and Mendell 2008). Using a similar analysis method (STEM algorithm) (Ernst and Bar-Joseph 2006), we grouped all mRNA genes from GSE178514 into 16 Profiles along with passage-dependent changes, and in Figure 2A, 6 Profiles with significance were obtained. The genes in Profiles 0, 2, and 3 were gradually downregulated while those in Profiles 12, 13, and 15 were gradually upregulated (Figure 2A). Considering that 29 miRNAs were downregulated and the relationship between miRNAs and mRNA was usually opposite, profiles 12, 13, and 15 including 210 genes were collected for downstream analysis (Figure 2B). In order to better understand these 210 genes, GO and KEGG function enrichment analysis was carried out. Consistently, we found that these genes participated in the ECM related pathways, such as “Mucin type O−glycan biosynthesis” and “ECM−receptor interaction” (Figure 2C), similar to the enrichment results of 29 miRNAs (Figure 1D). Moreover, these 210 genes also participate in the “Cellular senescence” pathway, indicating their potential function in regulating *in vitro* senescence of MSCs (Figure 2C). In addition, GO and KEGG enriched terms also indicates that these genes play a role in “HIF−1 signaling pathway,” “response to hypoxia,” and “response to oxygen levels” (Figures 2C, D).

### Determination of seven miRNA targets by target prediction analysis

**FIGURE 3:**
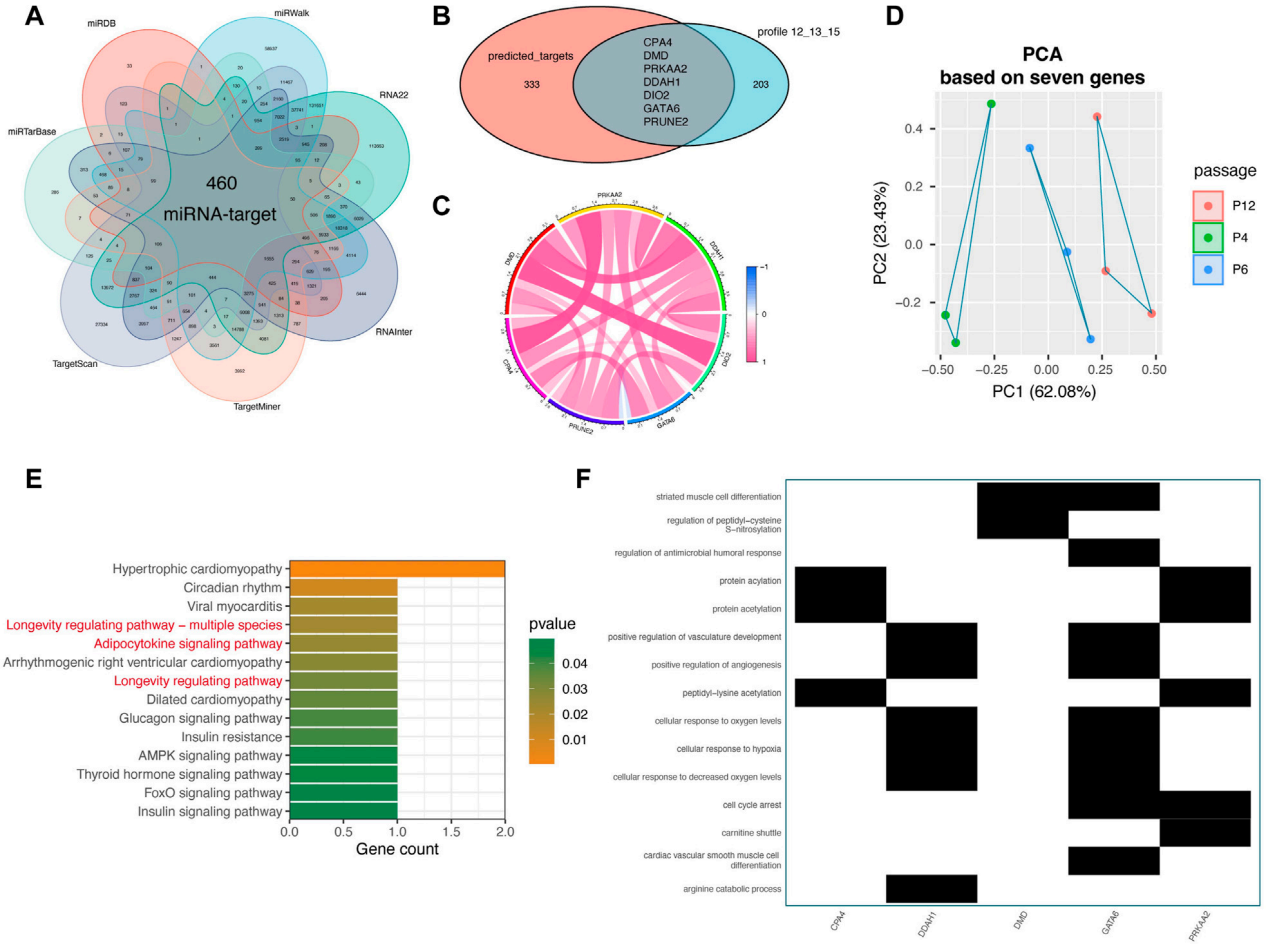
Target prediction analysis for 29 miRNAs and function analysis of key targets. **(A)** Intersection analysis of potential targets from seven miRNA target prediction databases for 29 miRNAs, including TargetScan (http://www.targets.can.org/vert._72/), miRTarBase (http://mirtarbase.mbc.nctu.edu.tw/php/index.php), miRDB (http://www.mirdb.org/), miRWalk (http://mirwalk.umm.uni-heidelberg.de/), RNA22 (https://cm.jefferson.edu/rna22/), RNAinter (http://www.rnainter.org/), TargetMiner (https://www.isical.ac.in/). **(B)** Intersection analysis of prediction targets and the 210 passage-specific mRNAs from mRNA Profile 12. **(C)** Correlation analysis among seven miRNA targets, containing CPA4, DMD, PRKAA2, DDAH1, DIO2, GATA6, and PRUNE2. **(D)** PCA analysis of MSCs based on seven target genes. “PCA” represents principal component analysis. **(E)** KEGG analysis of seven target genes. The top notable terms are displayed. **(F)** GO analysis of seven target genes.

Since 29 miRNAs were downregulated and 210 mRNAs were upregulated during MSCs passage, we then studied the regulatory relationship between these miRNAs and mRNAs. Seven target prediction database, including TargetScan (http://www.targets.can.org/vert _72/) (Lewis, et al., 2003), miRTarBase (http://mirtarbase.mbc.nctu.edu.tw/php/index.php) (Chou, et al., 2018), miRDB (http://www.mirdb.org/) (Chen and Wang 2020), miRWalk (http://mirwalk.umm.uni-heidelberg.de/) (Sticht, et al., 2018), RNA22 (https://cm.jefferson.edu/rna22/) (Miranda, et al., 2006), RNAinter (http://www.rnainter.org/) (Kang, et al., 2022), TargetMiner (https://www.isical.ac.in/) (Bandyopadhyay and Mitra 2009), were leveraged to predict the targets for these miRNA. After intersection of all “miRNA-target” pairs in these seven databases, 460 overlapped “miRNA-target” pairs were obtained (Figure 3A). Then, we intersected the prediction targets and the profiles with 210 gradually expressed mRNAs (Figure 3B). Finally, seven miRNAs (has-mir-454-3p, has-mir-196b-5p, has-mir-130b-5p, has-mir-1271-5p, has-let-7i-5p, has-let-7a-5p, and has-let-7b-5p) and related seven targets (PRUNE2, DIO2, CPA4, PRKAA2, DMD, DDAH1, and GATA6) were considered as hub miRNAs and targets which are critical for the maintenance of MSCs during the long-passages *in vitro* (Figure 3B).

As displayed in Figure 3C, these seven upwardly expressed and miRNA-regulated targets were highly correlated with each other, implying that that they may play a potential role together (Figure 3C). Notably, the PCA analysis based on only these seven genes vividly displayed that the *in vitro* passages of MSCs, P4, P6, and P12, were distributed following the principal component 1 (PC1) (accounting for 62.08%) (Figure 3D), confirming that these seven genes play a key role in regulating *in vitro* expansion of MSCs. Parallel to the aforementioned enrichment result s of 210 genes and 29 miRNAs, respectively, we found that GO and KEGG analyses of the seven genes showed that they also get involved in “Adipocytokine signaling pathway,” “Longevity regulating pathway,” “cellular response to oxygen levels”, and “cellular response to hypoxia” (Figures 3E, F). In general, seven hub miRNAs (has-mir-454-3p, has-mir-196b-5p, has-mir-130b-5p, has-mir-1271-5p, has-let-7i-5p, has-let-7a-5p, and has-let-7b-5p) and seven targets (PRUNE2, DIO2, CPA4, PRKAA2, DMD, DDAH1, and GATA6) were identified as important regulators in the *in vitro* expansion changes of MSCs.

### Construction of a “TF-miRNA-target” network to evaluate MSCs expanded *in vitro*

**FIGURE 4:**
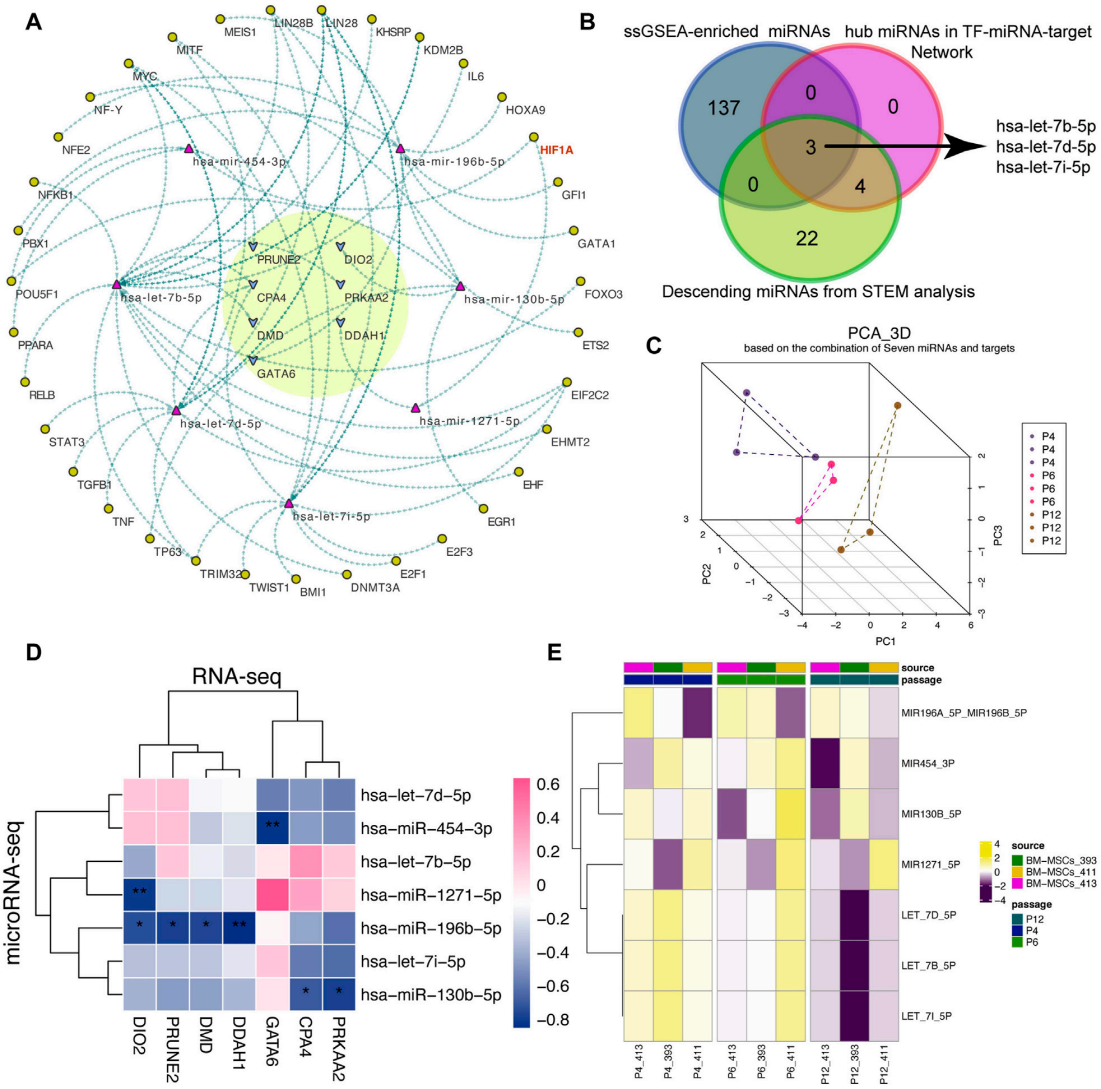
Construction of the “TF-miRNA-target” regulatory network. **(A)** A “TF-miRNA-target” regulatory network. The yellow circle highlights the hub targets. Arrows are the potential regulatory direction between linked nodes, including TFs, miRNAs and targets. **(B)** Venn plot of the overlapped miRNAs among ssGSEA-enriched miRNAs, hub miRNAs in network and descending miRNAs predicted from STEM analysis. And ssGSEA represents single sample gene set enrichment analysis. **(C)** 3D PCA analysis of MSCs according to seven miRNAs. 3D PCA: three-dimension principal analysis. **(D)** Correlation analysis between seven miRNAs and seven targets. **(E)** Activity score of the predicted miRNAs from ssGSEA algorithm.

Considering that TF also participate in the cell fate of MSCs, we tried to sort out the TFs that regulated the seven miRNAs by using the experimentally validated “TF-miRNA” pairs from TransmiR v2.0 database (Tong, et al., 2019), and thereafter constructed a “TF-miRNA-target” network related to the stemness and senescence of MSCs during passage (Figure 4A). Interestingly, several TFs including NFKB1, HIF1A, MYC, TGFB1, and IL6 are highly consistent with the above enrichment results. Therefore, we hypothesized that the “TF-miRNA-target” network used for *in vitro* expansion of MSCs may play a pivotal role in regulating MSCs cell fate, especially its cellular characteristics associated with stemness maintenance and senescence during the passages.

Next, we utilized the ssGSEA algorithm to calculate the miRNA activity score of the 210 upwardly expressed genes to obtain the potential miRNA-related regulon score and tried to validate the reliability of the miRNAs in the established network. After differential expression analysis, we found that the difference between P4 and P12 was more significant than P4 and P6 (Supplementary Figure S3). Using the Venn plot in Figure 4B, we found that members of the let-7 family can intersect under three different analysis methods, indicating that let-7 family may be significantly involved in the changes of MSCs cultured *in vitro*. The PCA algorithm based on seven miRNAs can also vividly depict the lineage-like changes of MSCs during cell passages along P4, P6, and P12 (Figure 4C).

Meanwhile, we also confirmed the negative correlation between miRNAs and targets using correlation analysis (Figure 4D) and the activity fraction of seven miRNAs calculated by ssGSEA algorithm also presented a downward activation trend along P4, P6, and P12 (Figure 4E).

### Validation of the seven passage-dependent genes

Although the “TF-miRNA-target” network we built had utilized various algorithms and collected experimentally verified “TF-miRNA” and “miRNA-target” pairs, whether these seven passage-specific genes could be used to evaluate the status of MSCs cultured *in vitro* still needs to be further verified by an external queue.

**FIGURE 5:**
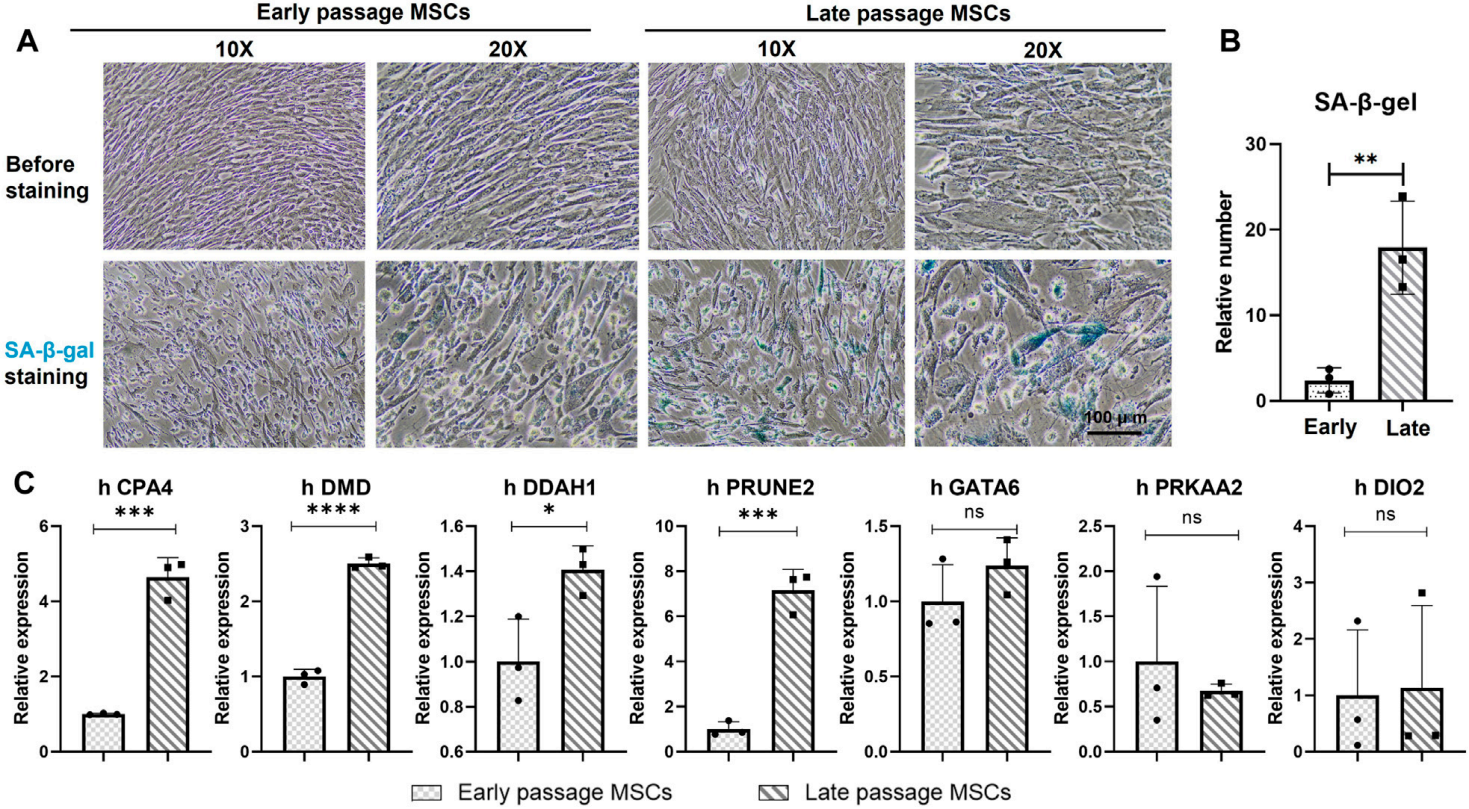
Validation of seven genes in early and late passages of MSCs. **(A)** Morphologies and the SA-**β**-Gal staining of early passage (P3) and late passage (P12) MSCs. Scale bar = 100 μm. Cells with blue dye inside of the cell plasma indicated as senescence cells (SA-**β**-Gal ^+^). **(B)** Quantitively analysis of the SA-**β**-Gal^+^cells in early passages and late passages MSCs. Data were generated from counting of positive numbers from three independence experiments. **(C)** qPCR verification of the seven genes, CPA4, DMD, PRKAA2, DDAH1, DIO2, GATA6 and PRUNE2 in early passage (P3) and late passage (P10 and P12) MSCs. *p < 0.05; **p < 0.01; ns: non-significant. N = 3.

Considering the variation of MSCs in different culture medium or systems (Supplementary Table S2), it isn’t logical to determine the stemness and senescence of MSCs solely on the numbers of passages. Application of MSCs morphological changes and SA- **β**- Gal intake was used to verify the senescence of early (P3) and late (P10 and P12) MSCs. In Figures 5A, B, in the serum free culture medium, MSCs of early passages maintained typical short spindle and fibroblast morphology, with transparency and homogenic morphology, and didn’t ingest SA-**β**-Gal after the overnight incubation. While, the late passage (P12) lost the typical homogenic morphology of MSCs, with larger and flatter morphological features, were also SA-**β**-Gal^+^, indicated the senescence of late passage (P12) under *in vitro* culture.

The mRNA of early passage (P3) and late passage (P10 and P12) MSCs were extracted to verify the expression of the seven genes during the expansion of MSCs *in vitro*. In Figure 5C, CPA4, DMD, DDAHq1, and PRUNE2 were significantly upregulated among the seven genes in the late passage MSC compared with the early passage MSC. Compared with the early passage, the expression levels of GATA6, PRKAA2 and DIO2 in the late passage MSCs also showed an upward trend. Although the difference wasn’t statistically significant, the overall expression trend of seven genes showed an upward trend during the senescence period of the late passage MSCs.

**FIGURE 6:**
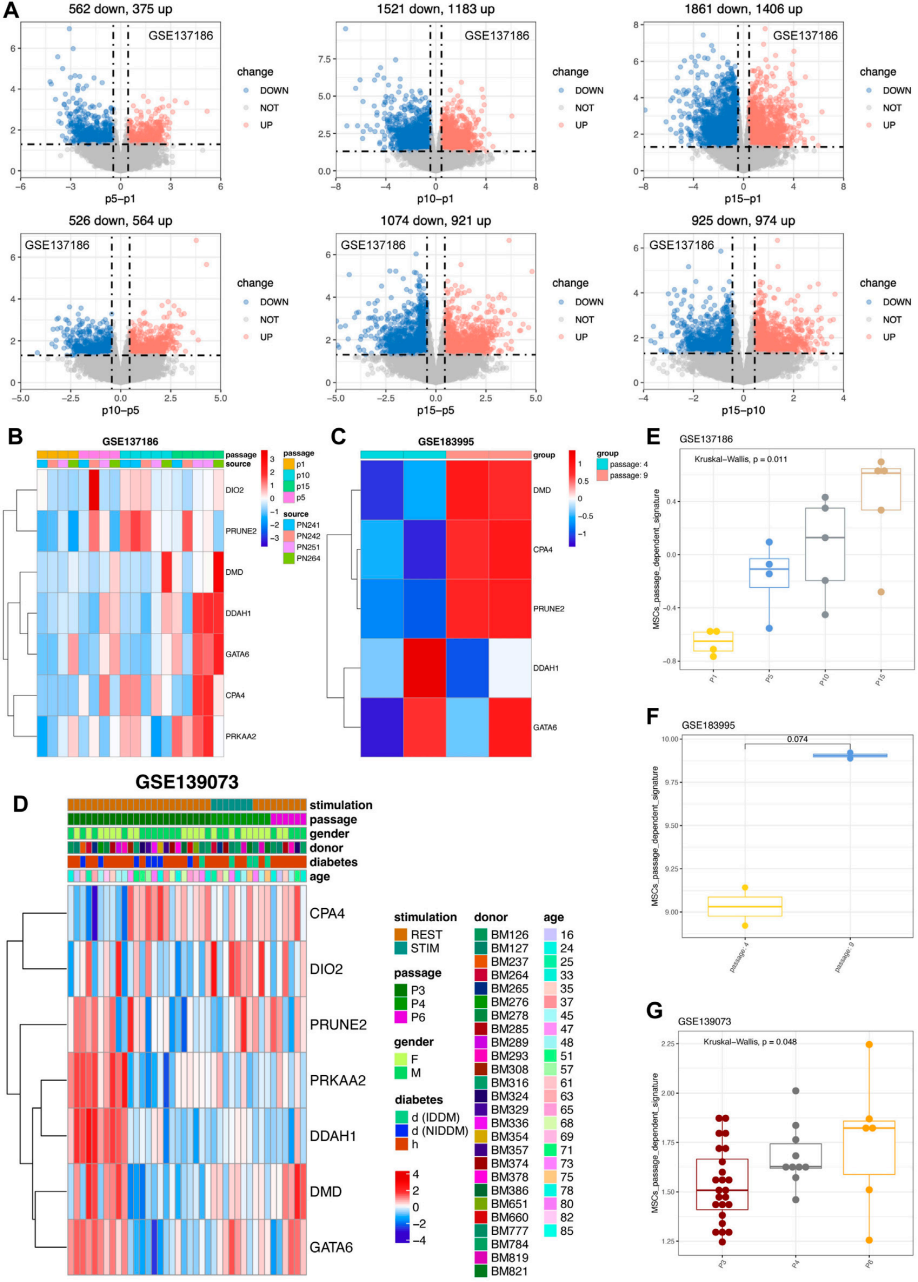
Validation of seven genes in external cohorts related to various passages of MSCs. **(A)** Differential analysis of various MSCs derived from different passages in GSE137186. The upregulated genes were in red color while the downregulated genes were in blue. **(B)** Expression pattern of seven genes in GSE137186, containing CPA4, DMD, PRKAA2, DDAH1, DIO2, GATA6 and PRUNE2. **(C)** Expression pattern of hub genes in GSE183995 and the statistical analysis were performed. **(D)** Expression pattern of seven genes in GSE139073. **(E)** Signature score of seven genes in GSE137186. **(F)** Signature score of hub genes in GSE183995. **(G)** Signature score of seven genes in GSE139073 and the statistical analysis were calculated.

We also did the verification with the datasets in public domain. By retrieving the expression profiles of cultured MSCs from P1, P5, P10, and P15 (Salerno, et al., 2020), and performed the differentially expression analyses of the MSCs from one to another passage. The results confirmed that MSCs had passage changes, and the difference was more significant after more than 10 passages, and this change was related to cell senescence of MSCs in the late passage period (Figure 6). To better estimate whether MSCs had undergone *in vitro* passage-specific changes, we adopted the ssGSEA algorithm to calculate the seven-gene signature score for MSCs cultured *in vitro*. Intriguingly, results distinctly showed that the passage-dependent score became gradually higher from P1, P5 to P10 and finally P15 (Figure 6E). On the other hand, although the comparison was not significant, the signature score was prone to be higher in P9 than in P4 (Figure 6F). And notably, the signature score increased gradually from P3, P4 to P6 (Figure 6G). Therefore, the seven-gene signature can be a powerful tool for detecting the changes of MSCs cultured *in vitro*.

With regard to the MSCs from young donors, results showed that six of the seven gene have tendency to highly express in P8 (Supplementary Figures S5A, B) and the seven gene signatures were also highly activated in P8 (Supplementary Figure S5C). From the perspective of old donor, six of the seven genes, including DMD, PRKAA2, DDAH1, DIO2, GATA6, and PRUNE2, were more likely to highly express in P8 MSCs (Supplementary Figures S5D, E). Parallelly, the seven gene signatures were also higher activated in old P8 MSC rather than P4 MSC (Supplementary Figure S5F). However, the Wilcoxon analysis didn’t show enough significance possibly owing to the passage of MSCs were much closer and less than 10, indicating the MSCs product still in a good manufacturing state. To sum up, the seven genes (PRUNE2, DIO2, CPA4, PRKAA2, DMD, DDAH1, and GATA6) were passage-dependent, especially when the number of passages exceeds 10.

### The seven gene signatures were also useful to evaluate the manipulated MSCs

**FIGURE 7:**
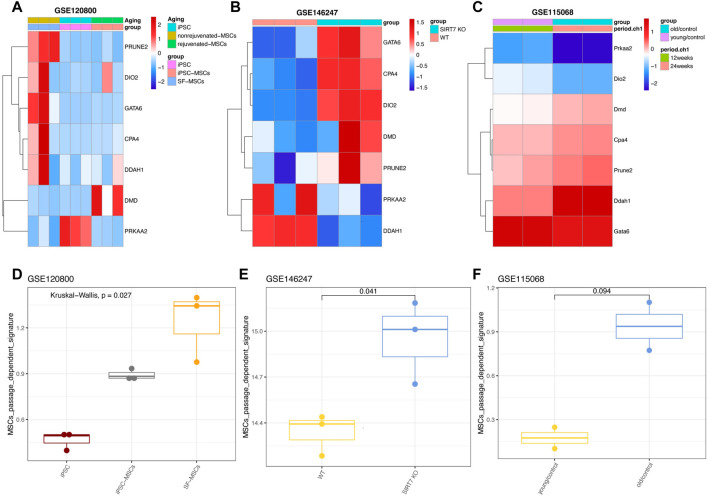
Expression patterns of seven genes in manipulated MSCs. **(A)** Expression pattern of seven genes in GSE120800 **(B)** Expression pattern of seven genes in GSE146247. **(C)** Expression pattern of seven genes in GSE115068. **(D)** Signature score of seven genes in GSE120800. **(E)** Signature score of hub genes in GSE146247. **(F)** Signature score of seven genes in GSE115068 with statistical analysis.

Next, we investigated whether the seven gene signatures (PRUNE2, DIO2, CPA4, PRKAA2, DMD, DDAH1, and GATA6) could be validated by MSCs that were generated from pluripotent stem cells (PSCs), such as embryonic stem cells (ESCs) or induced pluripotent stem cells (iPSCs). These PSC-derived MSCs had shown enormous application potentials in treating diseases as they can overcome the hurdle of replicative senescence associated with the *in vitro* expansion of primary cells. To our surprise, we found the expression of seven genes of iPSCs-derived MSCs tended to be lower than that of ordinary MSCs, which indicating that the MSCs were rejuvenated as the authors have experimentally validated (Figure 7A) (Jiao, et al., 2021). Using another dataset (GSE146247) (Bi, et al., 2020), we found that most of the seven genes were highly expressed in SIRT7-knock-out senescence-induced MSCs (Figure 7B). This result was in line with the author: SIRT7 is decreased during the senescence of MSCs, and its overexpression reverses the early senescence phenotype of MSCs (Bi et al., 2020).

We also tried to use the mouse data set (GSE115068) to test whether the seven gene signatures can be used for *in vivo* senescence of MSCs (Taketani, et al., 2019). Consistently, most genes also tended to be expressed in MSCs from old mice, however, the differences between the young and the older weren’t statistically significant (Figure 7C). In parallel, the passage-dependent signature was also calculated, and results showed that our seven gene signatures can vividly display the changes of MSCs under different gene manipulation to reprogram or rejuvenate them (Figures 7D, E).

### The passage-dependent signature has limited predictive effect on senescence of MSCs *in vivo*

A recent study on human MSCs reported that donor age had little influence on the phenotype and functional characteristics of MSCs (Andrzejewska, et al., 2019). In addition, there is no significant difference between the characteristic scores of old mice and young mice. We assume that our passage-dependent signature might not be suitable for the senescence of MSCs *in vivo* (Figure 7F). To test this hypothesis in an external cohort, we collected another dataset (GSE25069) containing MSCs from young and old mouse (Nodari, et al., 2021). The results showed that most of the passage-specific genes in MSCs obtained from 12-month-old mice rather than 1-month-old were prone to be upregulated, but the Wilcoxon analysis wasn’t significant (Supplementary Figures S6A, B). And the signature score was also highly activated only in 12-month-old mice (Supplementary Figure S6C).

Considering the possible influence of the heterogeneity of MSCs on the accuracy of gene feature prediction, we collected a single cell sequencing dataset (GSE145477), including 25, 919 single MSCs from mice at 1, 1.5, 3, and 16 months (Zhong, et al., 2020; Zhong, et al., 2022) (Supplementary Figure S7). Similarly, at the single cell level, no age dependent trend was found for most genes (Supplementary Figure S8). The ssGSEA algorithm further confirms this point. The algorithm is usually used to analyze gene activity scores. Compared with the early age, the passage dependence characteristics of MSCs aged 16 months don’t show a trend of increasing activity (Supplementary Figures S9A, B). Therefore, the passage-dependent signature contributes less to the age of MSC status assessment *in vivo*, which may be due to the dilution and interference of MSC complex and comprehensive microenvironment signals.

### Reconfirming the robustness of seven gene signatures in estimating passage-dependent characteristics of MSCs

**FIGURE 8:**
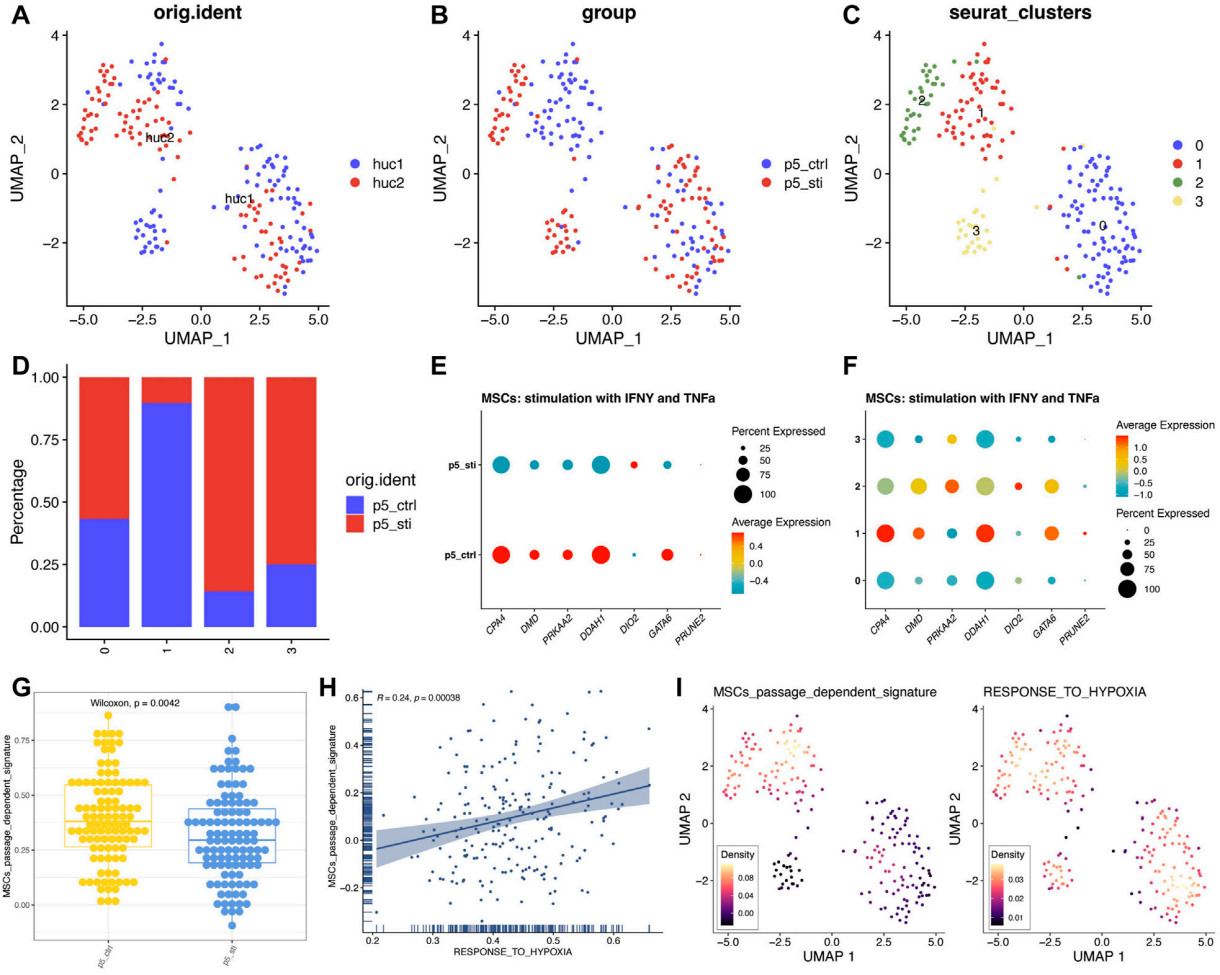
High expression trend of seven genes of MSCs treated with IFNγ and TNFα. **(A)** UMAP plot of MSCs from different sources. **(B)** UMAP plot of MSCs from different groups. **(C)** UMAP plot of MSCs from different clusters. **(D)** Detailed proportion of MSCs in different clusters and groups. **(E)** Expression pattern of seven genes in different groups. **(F)** Expression pattern of seven genes in different clusters. **(G)** Signature score of seven genes in different groups. **(H)** Correlation analysis between different signatures. **(I)** UMAP plot of different signatures.

Lastly, we extended the verification of the seven gene signatures developed by the “TF-miRNA-target” network at the single cell level with a scRNA-seq dataset (GSE117837), which was generated by stimulating (IFN γ+ TNF α) and was sequenced by Smart-seq2 technology (Huang, et al., 2019). Since our signature was developed on P4-P10 MSCs, we used strict criteria to screen scRNA sequence data to obtain P5 MSCs pre- and post-stimulation (Supplementary Figure S10). In total of 211 single cells were collected and clustered with the top 2,000 gene with differences (Figures 8A, 8B; Supplementary Figure S11). The cluster two and three were mainly composed of MSCs treated with IFNγ and TNFα, while cluster one mainly composed of MSCs with no treatment (Figures 8C, D). Then, we found that all the seven genes can be inhibited by IFNγ and TNFα treatment (Figures 8E, F; Supplementary Figure S12). Seven gene signatures can also be inactivated by IFNγ and TNFα stimulation (Figure 8G). Considering IFNγ and TNFα treatment can enhance the immunomodulatory, protein-secreted, anti-aging ability of MSCs (Huang, et al., 2019), we reassured that seven gene signatures can help us judge the status of MSCs cultured *in vitro*. In particular, we also found that the passage-dependent signature activity was positively correlated with “Response to hypoxia” pathway, which was consistent with the GO and KEGG enriched results of seven genes (Figure 8H). And the passage-dependent signature activity and the “Response to hypoxia” pathway activity were mostly from same cells (Figure 8I). Thus, the seven gene signatures were passage-dependent, and the oxygen level may be related to the changes of these genes.

## Discussion

Mesenchymal stem/stromal cells are a resource for autologous and allogeneic cell therapy for immunology-related and regenerative medicine. At current, more than a dozen MSC drugs have been approved by FDA to treat diseases including Acute graft versus host disease (aGVHD) (Temcell®), complex perianal fistula in adult patients with acute inactive/mildly active Crohn’s disease (Alofisel®), severe limb ischemia due to Buerger’s disease and atherosclerotic peripheral artery disease (Stempeucel®) etc. The *in vitro* expansion condition of MSCs has been greatly developed in the past two decades (Supplementary Table S2), which has enabled MSCs to be obtained from variety sources and prepared with standard produces, making the MSCs as the current most promising type of stem cells for other end-stage diseases such as heart failure or liver diseases.

However, the long-term culture of preparation evokes continuous changes to MSCs. And increasing numbers of studies adapted the scRNA-seq and other multiple-omics assays to reveal the heterogeneity and subpopulations in the single-prepped MSCs, which might cause the therapeutic consequences. Therefore, it is highly demanded for set of reliable parameters which can be used for the quality control of MSCs products. In a study by Wagner, by comparing the genetic signature of MSCs at passage 2-11, it has been proved that the gene expression changes, and epigenetic modifications are continuously acquired during replicative senescence (Wagner et al., 2010).

Previously, we established a specific “TF-miRNA-Target” regulatory network to investigate the underlying mechanisms of the lineage maturation of hepatic stem cells into mature cells (Wang X. et al., 2021). This “TF-miRNA-Target” was proved to be validated to predict the genes or the signaling pathways which are related to the maturation of hepatocytes from various sources. In this study, we identified the passages-related genes and miRNAs that may regulate MSCs during the *in vitro* expansion by analyzing transcriptome sequencing of MSCs that have been passed through multiple passages. These discovered genes and miRNAs not only revealed the molecular mechanism of MSCs senescence *in vitro*, but also provided useful tools for the quality control of MSCs during the preparation under different expansion conditions.

Among all the parameters, cell senescence is mainly responsible for the decreased activity and function of tissue derived MSCs and is considered to be a major challenge for MSC therapy and tissue engineering. Wagner et al. addressed the replicative senescence of human MSCs, and had found that genes related to cell cycle, DNA replication and DNA repair are significantly downregulated in late passages. And these changes are not restricted to later passages but are continuously acquired with increasing passages (Wagner et al., 2008). Ryu et al. (2008) had reported that they conducted microarray analysis to compare the transcriptomic profiles of early (P7) and late passage (P15) MSCs and finally recognized 338 senescence-related genes. And a recent study has compared the changes of premature MSCs with MSCs using multi-omics sequencing to better understand the maturation and aging of MSCs (Liu et al., 2022).

In the current study, we used a variety of sequencing data to identify seven key genes (PRUNE2, DIO2, CPA4, PRKAA2, DMD, DDAH1, and GATA6) that regulate passage-committed and aging-related changes in the *in vitro* cultured MSCs. Among them, GATA6 is an added key regulator in aging MSCs, which controls the hedgehog signal and FOXP1 pathway to regulate cell senescence and senescence-related activities (Jiao, et al., 2021). Studies of Prune homolog 2 (PRUNE2) in normal stem cells weren’t well reported, yet it`s expression or silencing in prostate cancer cells decreased and increased cell proliferation, respectively. Thus indicated PRUNE2 as a tumor suppressor gene in human (Salameh et al., 2015). While a study by Artsi indicated that in bone marrow adipose tissue, over expression of a gene named Sirtuin1 (Sirt1) increased Foxc2, Pgc1α, Dio2, Tfam, and Cyc1 expression, which play a key effect in the metabolism in both murine and human (Artsi et al., 2019). Carboxypeptidase A4 (CPA4) is a member of the metallo carboxypeptidase family which have been identified to be involved in cancer biology and insulin sensitivity (Gao et al., 2020). As the matter of fact that CPA4 promote the proliferation or the progression of tumor by activating the PI3K-AKT-mTOR signaling might be the reason that it has been sorted out from the differential genes of early passages MSCs and late passage MSCs with senescence in our study. These findings might be crucial for enhancing the understanding of MSC aging and the diseases related to MSC aging as well as providing insight for developing pharmacological strategies for improving cell state of MSCs.

Several signaling pathways had been found changing significantly in the late passage MSCs versus MSCs in early passages, many were associated with ECM. *ex vivo* MSCs are surrounded by ECM composed of collagen, adhesion proteins, proteoglycans and growth factors (Moore and Lemischka 2006). MSC living in ECM not only receives signals from ECM, but also affects ECM by secreting ECM components and by proteolytic modification of protein and growth factors in ECM (Behonick and Werb 2003). Aged MSCs themselves can also change the composition of the ECM. An example of how ECM can alter the characteristics of stem cells is the recent discovery that human embryonic stem cells would lose their ability to regenerate when exposed to an aging ECM (Carlson and Conboy 2007). Therefore, ECM-related pathways might participate in regulating the aging and anti-aging features of MSCs, which is in line with our result that the ECM-related (Liu et al., 2022) pathways might be responsible for the changes of *in vitro* cultured MSCs (Figures 1D, 2C; Supplementary Figure S2). The changes of ECM deposition of MSCs during culture is still under investigated. In our study, by compared the MSCs in culture at different passages, we had revealed a consistent ECM structure organization and collagen-containing ECM formation during the maintenance of the MSCs in the culture (Figure 2).

Other signaling pathways related to the stability of MSCs *in vitro* were oxygen-related pathways (Figures 1D, 2C, 3F), which has highlighted the involvement of metabolic regulation during replicative senescence of MSCs. Similar to what has encountered for the MSCs *in vivo*, cultured MSCs *in vitro* face a plethora of signals which requires the metabolic plasticity to be able to maintain stable during expansion. It has been proved that alternations of cellular nicotinamide adenine dinucleotide (NAD+/NADH) redox balance during the expansion of MSCs causes the cellular senescence of MSCs, while re-balancing the NAD+/NADH ratio can enhance the activity of sirtuin-1(Sirt-1) in high-passage-MSCs to rejuvenates senescent hMSCs (Yuan et al., 2020). A proteomics study of the human MSCs cultured in variety conditions for short term (1 week) or long-term (1 month) revealed the lysosome, autophagy and post-translational protein modifications were most critical for the aging of MSCs (Wang et al., 2022). Autophagy is used to be considered as a suppress factor of MSC senescence is now proved to promote the MSC senescence by stimulating the producing of senescence-associated secretory proteins (SASP) (Rastaldo et al., 2020). In muscle, basal autophagy was proved to be essential to maintain the muscle stem cell quiescent. Losing the capacity of autophagy causes cellular senescence of satellite cells, while regaining of autophagy restores the functions of satellite cells (García-Prat et al., 2016). In human MSCs, cultivation under low oxygen level has been proved as a sufficient approach to reverse cellular senescence, mainly by upregulating the expression of autophagy related genes at the transcriptional level (Ratushnyy et al., 2020).

There are several studies had shown that MSC quality declines with age, and continuous subculture leads to changes in cell morphology, enlargement and ultimately senescence. Among those studies, Stolzing et al. had compared MSCs from bone marrow is donors from age 5 to 55 for their colony formation assay and the proliferative rate and found an overall age-related decline in BM MSC “fitness” (Stolzing et al., 2008). The markers of senescence included ROS levels, p21 and p53 were found elevated during along the aging, which indicating there are correlations of the senescence of MSCs with the ages of the donors.

Another interesting feature of aged MSCs is that cells tend to differentiate towards the fat lineage. It has been reported that cell senescence promotes lipogenesis of mouse MSCs and inhibits osteogenesis (Moerman et al., 2004). And the volume of adipose tissue in the bone marrow increases with donor ages and/or diseases such as osteoporosis (Justesen et al., 2001). A study carried out by Moravcikova had carefully investigated the changes of cell surface markers of senescence MSCs during *in vitro* culture. The result also indicated that the early senescence of MSCs were correlated with changes of CD markers which then impact the differentiation potential of MSCs (Moravcikova et al., 2018). Congruently, our enrichment analysis of seven genes also pointed out the “Adipocytokine signaling pathway,” indicating that the seven-gene signature was related to the senescence of MSCs again (Figure 3E).

There are some clarifications and limitations need to be noticed in current study. Here we have identified seven miRNAs and mRNAs which can be used to monitor the replicative senescence of MSCs in culture. Although in most datasets, the expression of the seven gene signatures is upregulation in MSCs that have been passaged for more than 10 times, it is impossible to simplify which passage of MSCs should be considered as early passage MSC or late passage MSC because of the wide range of culture conditions used in different facilities (Supplementary Table S2). Therefore, it is recommended to use a direct experimental senescence assay, i.e., SA-**β**-Gal staining, to confirm replicative senescence once the expression level of the seven gene signature is detected.

Another issue that needs to be clarified is the application of seven gene signatures as markers in the quality control of cultured MSCs. Ideally, when the culture conditions are appropriate, all seven genes should be low expressed in the late MSCs and maintain the same level as in the early MSCs (P1-P3, no SA-**β**-Gal intake). However, as shown above, depending on the number of passage or the culture conditions applied, it isn’t surprising to find that not all seven genes are upregulated in MSCs with a senescence property. Similar to other studies on genetic characteristics that predict cell or tumor status (Wang et al., 2021; Zhou et al., 2021), it is logical to take seven gene signatures as a set. Compared with each individual, its overall expression trend should be more accurate.

As important as it is of the seven gene signatures for the prediction of replicative senescence of cultured MSCs, there are some limitations during the prediction of the targets of miRNAs. By matching the expression trends with the mRNAs in Figure 2A, some miRNAs may be excluded. This may cause the key signal pathways revealed by KEGG/GO to be insignificant, such as several metabolic pathways (also overlapping with Figure 2), but the number of genes is extremely low.

In addition, we chose the stemness maintenance and the senescent as the two key criteria of the quality control of MSCs in culture. However, other than the ECM and the stemness related signaling pathways, TGF−beta signaling pathway, Hippo signaling pathway and FoxO signaling pathway were also enriched in the GO and KEGG analysis. Those signaling pathways are closely related to the functions of MSCs related to their paracrine biological activities which are one of the most important features for MSCs as a therapeutic cell product. Although the signaling pathways related to the stemness has covered the differentiation potential and therapeutic potential of MSCs, the models we established here in this study can be further trained to be able to evaluation the MSCs product more accurately.

To sum up, the seven gene signatures (PRUNE2, DIO2, CPA4, PRKAA2, DMD, DDAH1, and GATA6) is a possible candidate marker of estimating the MSC *in vitro* senescence. And oxygen-related and ECM-related pathways might play pivotal roles in regulating the aging and anti-aging characters of MSCs. However, how to judge the MSC *in vivo* senescence remains a challenge due to its limited changes, and more work needs to be done in this area. And only seven genes were identified might not cover the whole characters of *in vitro* senescence. In particular, more characterization of MSCs *in vivo* is needed also in its native environment.

## Conclusion

In conclusion, our study identified potential miRNAs (has-mir-454-3p, has-mir-196b-5p, has-mir-130b-5p, has-mir-1271-5p, has-let-7i-5p, has-let-7a-5p, and has-let-7b-5) and genes (PRUNE2, DIO2, CPA4, PRKAA2, DMD, DDAH1, and GATA6) related to MSC *in vitro* expansion, thus established a “TF-miRNA-target” regulatory network, in order to better describe the changes in cell status and the occurrence of replicative senescence of MSCs cultured *in vitro*. Other researchers have verified the regulation between miRNAs and various MSCs-related TFs through experiments. Among the seven genes that were gradually upregulated, GATA6 was confirmed to be involved in the regulation of MSCs senescence. Future research not only needs to identify the specific functions of related miRNAs and genes in MSCs, but also needs to dissect the “TF-miRNA-target” regulatory network on MSCs during aging. More importantly, *in vitro* and *in vivo* studies are needed to determine the prerequisites for improving the efficacy of regenerative medicine MSCs products. In addition, consistent with the previous conclusions, replicative senescence *in vitro* rather than *in vivo* has a greater impact on the characteristics of MSCs. Long-term of *in vitro* culture and expansion will damage the regeneration function of MSC in later passages. Therefore, the passage numbers of MSCs should be strictly controlled for their preclinical and clinical application. The treatment preferably requires a large number of minimally expanded cells with ideal efficacy under the standard and well monitored protocol.

## Data Availability

The datasets presented in this study can be found in online repositories. The names of the repository/repositories and accession number(s) can be found in the article/Supplementary Material.
