# Supplementary material for: A passage-dependent network for estimating the in vitro senescence of mesenchymal stromal/stem cells using microarray, bulk and single cell RNA sequencing
Source: Front Cell Dev Biol. 2023 Feb 7;11:998666. doi: 10.3389/fcell.2023.998666 (PMC9941187; doi:10.3389/fcell.2023.998666)
Supplement: Supplementary file 1 [file DataSheet1.PDF]

A passage-dependent network for estimating the *in vitro* senescence of mesenchymal stromal/stem cells using microarray, bulk and single cell RNA sequencing

Yong Yang[1†], Wencheng Zhang[2,3,4†], Jingxian Yang[5], Xicheng Wang[2,3,4], Yangyang Cui[2,3,4,6], Haimeng Song[2,3,4], Weiping Li[7], Wei Li[8], Le Wu[9], Yao Du[1], Zhiying He[2,3,4*], Jun Shi[1*], Jiangnan Zhang[1*]

Supplementary data

Figure S1. Detailed regulatory mechanism in KEGG_hsa_04550 pathways (Signaling pathways regulating pluripotency of stem cells). The different color of each target indicates the potential number of microRNAs that they might be shared. The KEGG pathway map was obtained from the KEGG database. Diana MirPath_v3 database was used to visualize hub genes.

KEGG_hsa04550

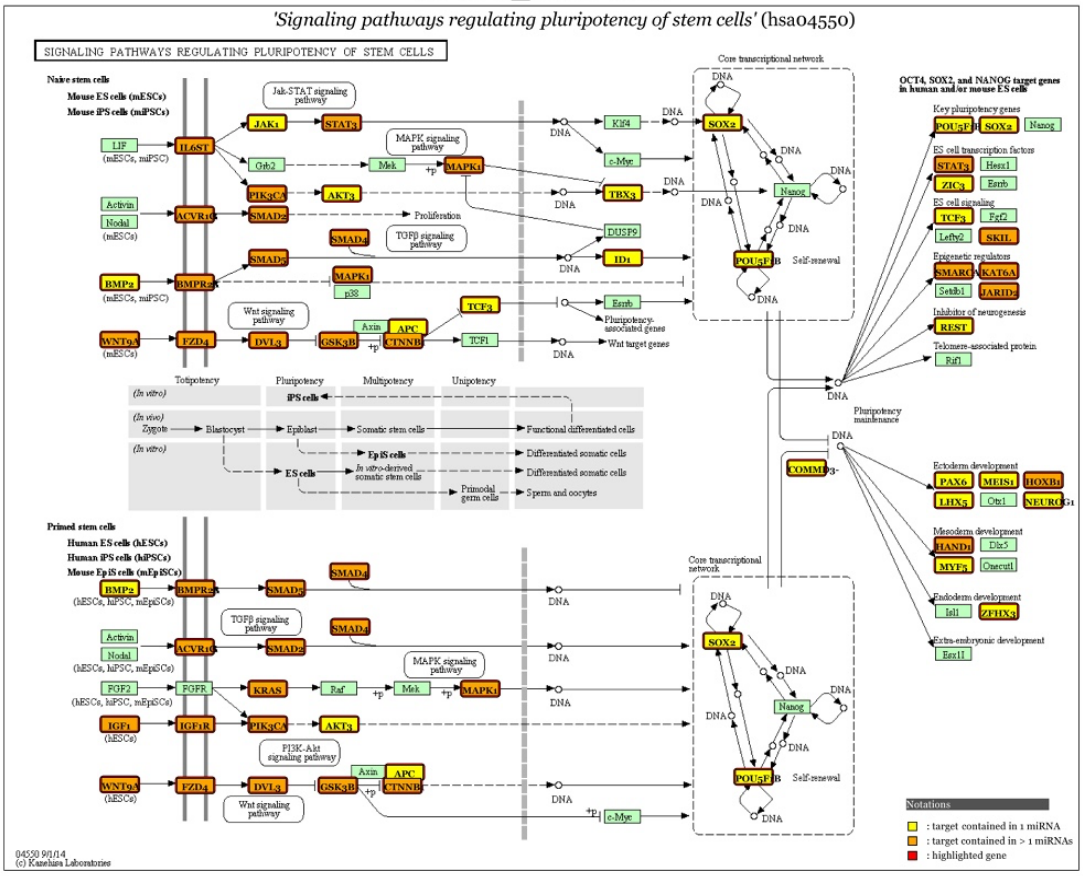

Figure S2. Detailed regulatory interaction of KEGG_hsa_04512 pathways (ECM-receptor interaction). The different color of each target indicates the potential number of microRNAs that they might be shared.

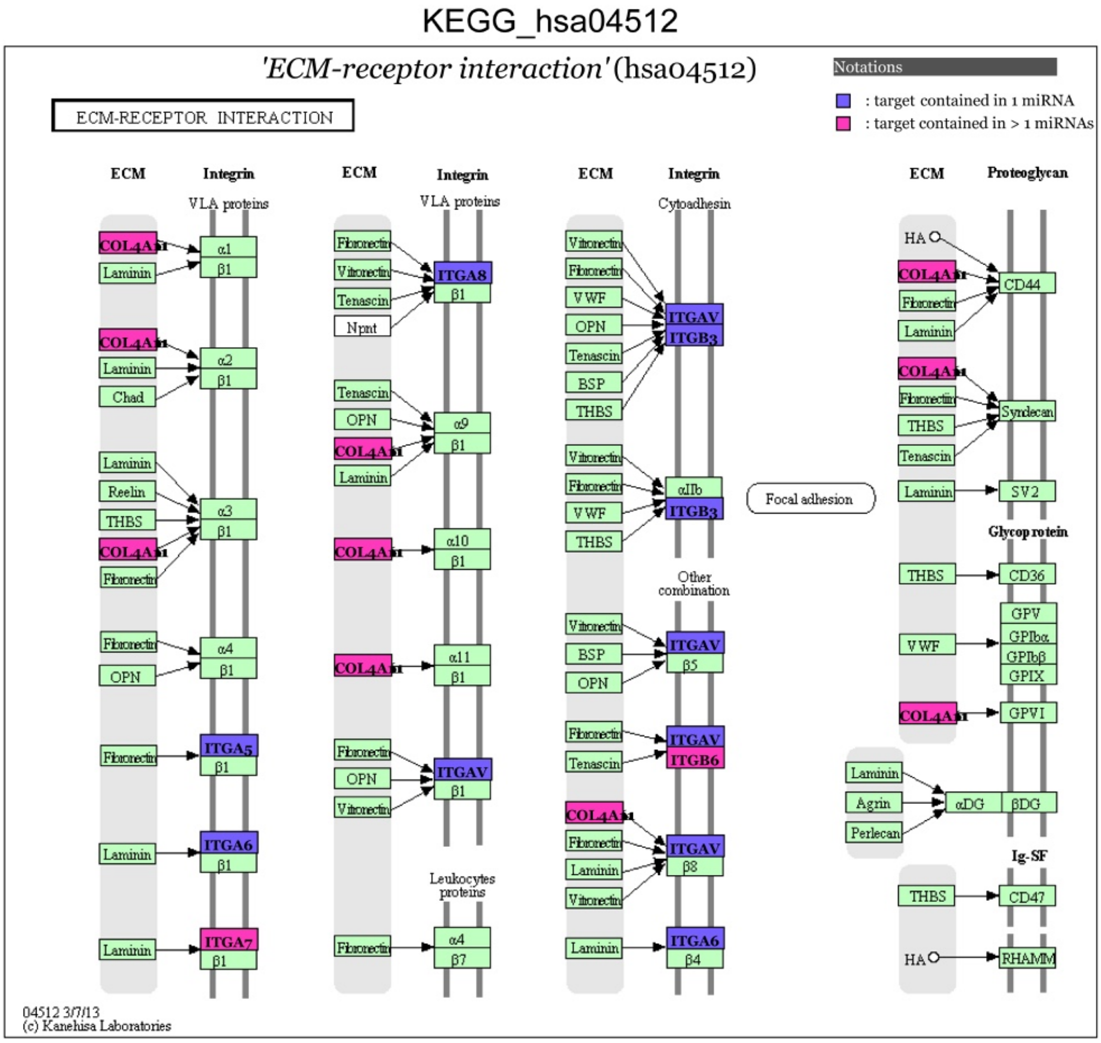

Figure S3. Differential analysis of ssGSEA-predicted microRNAs. Each spot indicates a single ssGSEA-predicted microRNA.

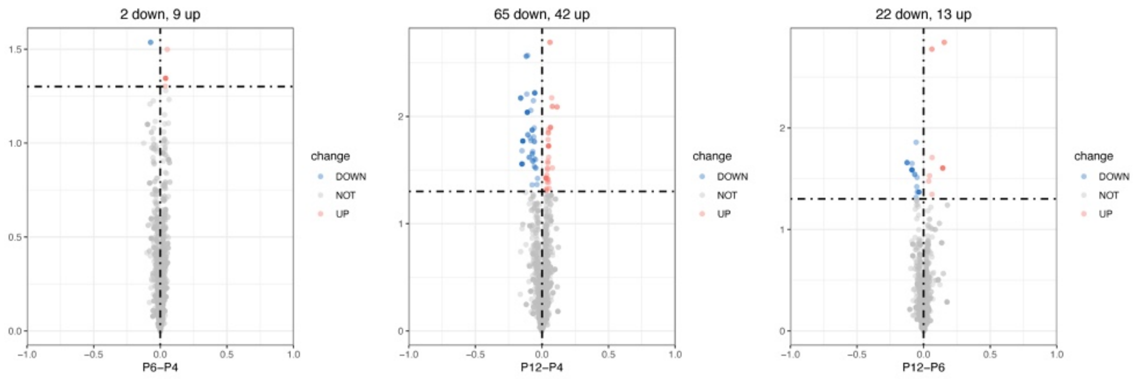

Figure S4. Expression pattern of the passage-specific genes in GSE110755. (A) Heatmap shows expression pattern of seven genes in GSE110755. Cluster analysis was adopted to show the expression of different genes. (B) Boxplot shows expression pattern of seven genes in GSE110755. KRUSKAL-WALLIS analysis was performed for the statistical analysis. (C) Boxplot shows signature scores in GSE110755.

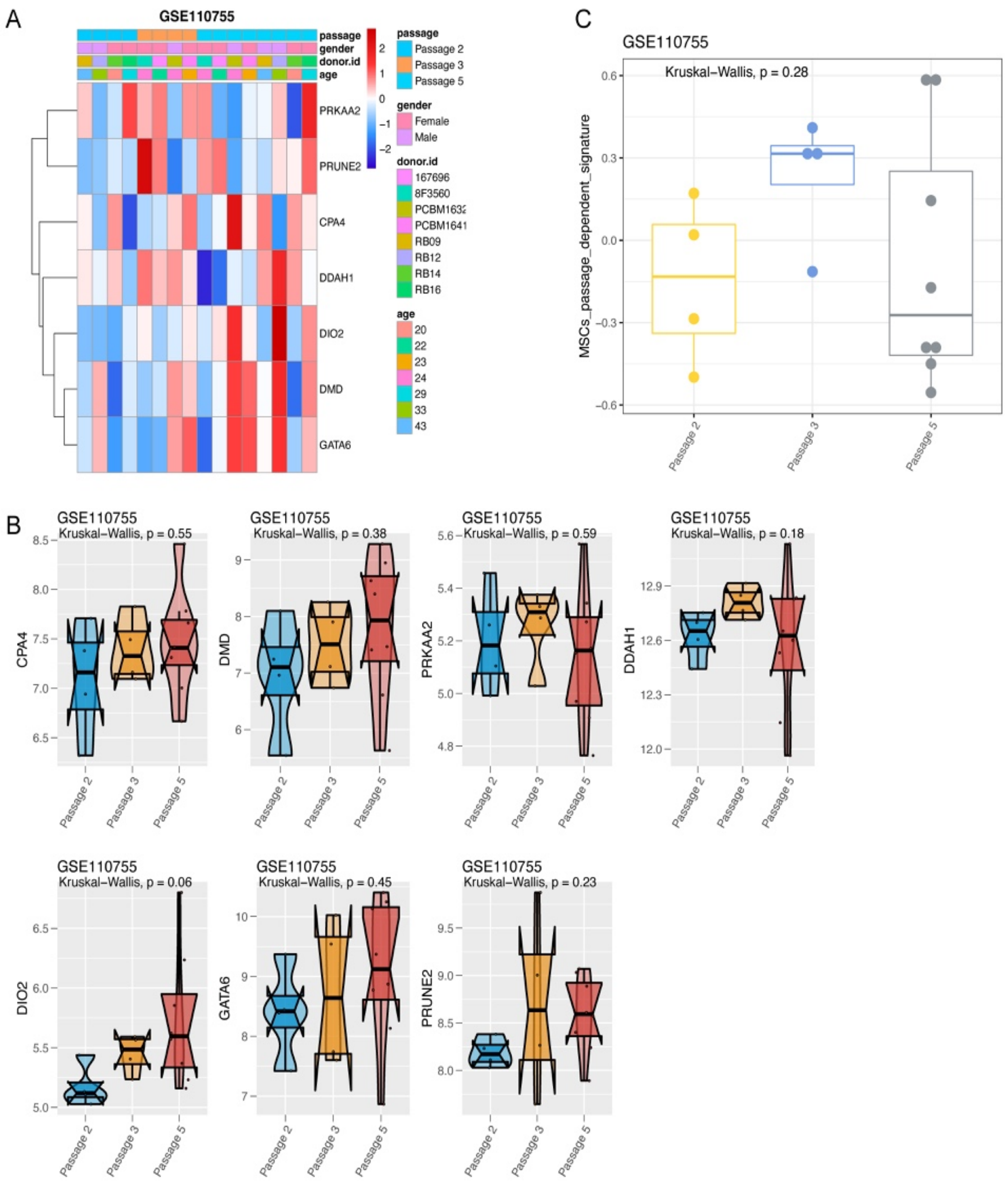

Figure S5. Expression pattern of the passage-specific genes in GSE39035. (A) Heatmap shows expression of the seven genes in young MSCs in GSE39035. (B) Boxplot shows expression pattern of seven genes in young MSCs in young MSCs in GSE39035. Statistical analysis (KRUSKAL-WALLIS analysis) was performed. (C)

Boxplot shows signature scores in young MSCs in GSE39035. (D) Heatmap shows expression of seven genes in old MSCs in GSE39035. Cluster analysis was performed to show the expression of different genes. (E) Boxplot shows expression pattern of seven genes in old MSCs in young MSCs in GSE39035. (F) Boxplot shows signature scores in old MSCs in GSE39035.

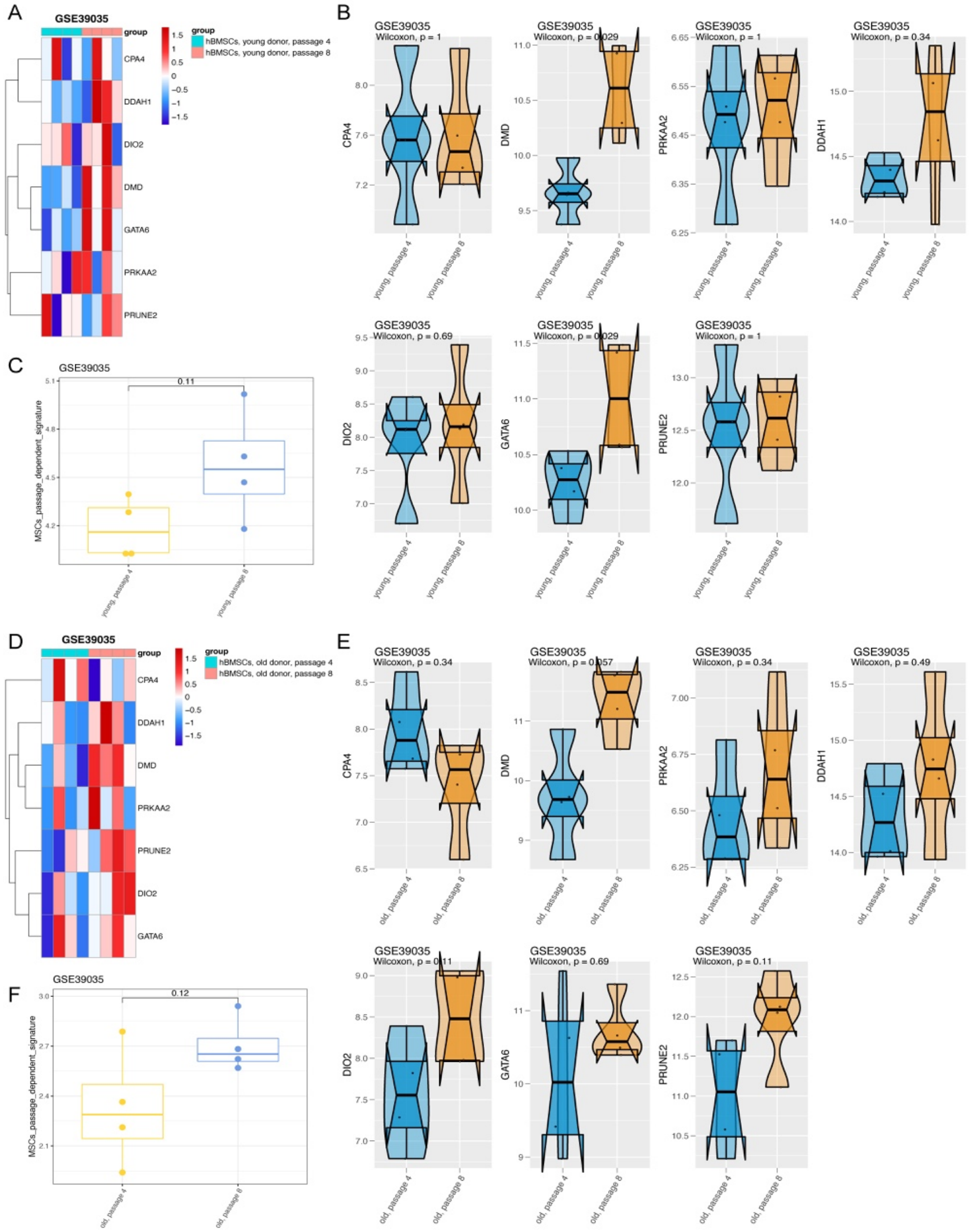

Figure S6. Expression pattern of the passage-specific genes in GSE25069. (A) Heatmap shows expression pattern of seven genes in GSE25069. (B) Boxplot shows expression pattern of seven genes in GSE25069. (C) Boxplot shows signature scores in GSE25069. WILCOXON analysis was used for the statistical analysis.

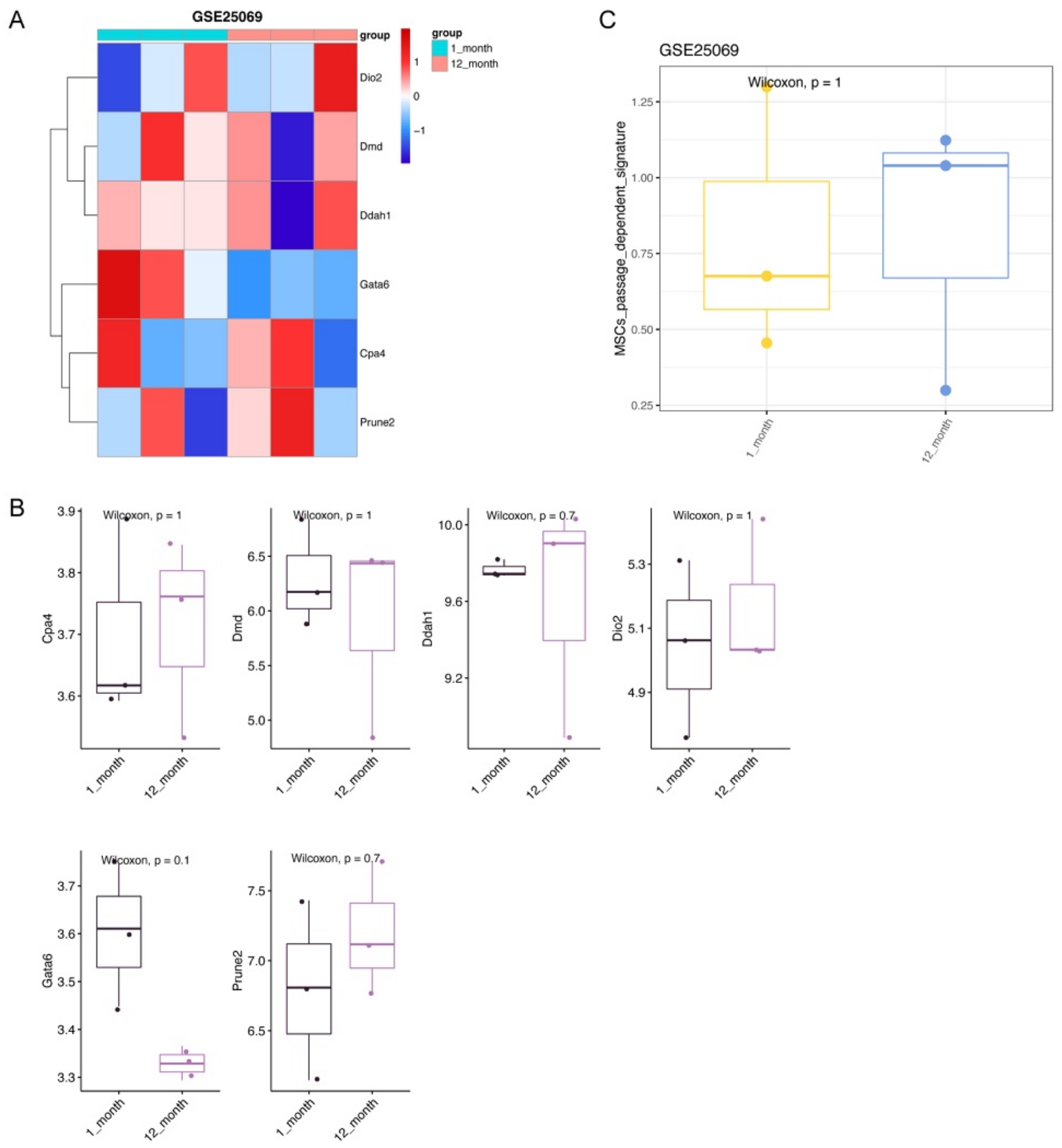

Figure S7. Single cell RNA-seq analysis of GSE145477. (A) Expression percentage of mitochondria genes in GSE145477 after filtering the single cells with stringent criteria. (B) Expression percentage of hemoglobin genes in GSE145477 after filtering the single cells with stringent criteria. (C) Expression percentage of ribosome protein genes in GSE145477 after filtering the single cells with stringent criteria. (D)(G) Expression levels of mitochondria genes in GSE145477 after filtering the single cells with stringent criteria. (E)(H) Expression levels of hemoglobin genes in GSE145477 after filtering the single cells with stringent criteria. (F)(I) Expression levels of ribosome protein genes in GSE145477 after filtering the single cells with stringent criteria. (J)(K) Expression pattern between mitochondria genes and ribosome protein genes. (L) Violin plot of total RNA read count (nCount_RNA), total gene count (nFeature_RNA), pHB (expression percentage of hemoglobin genes), pMT (expression percentage of mitochondria genes) and pRP (expression percentage of ribosome protein genes).

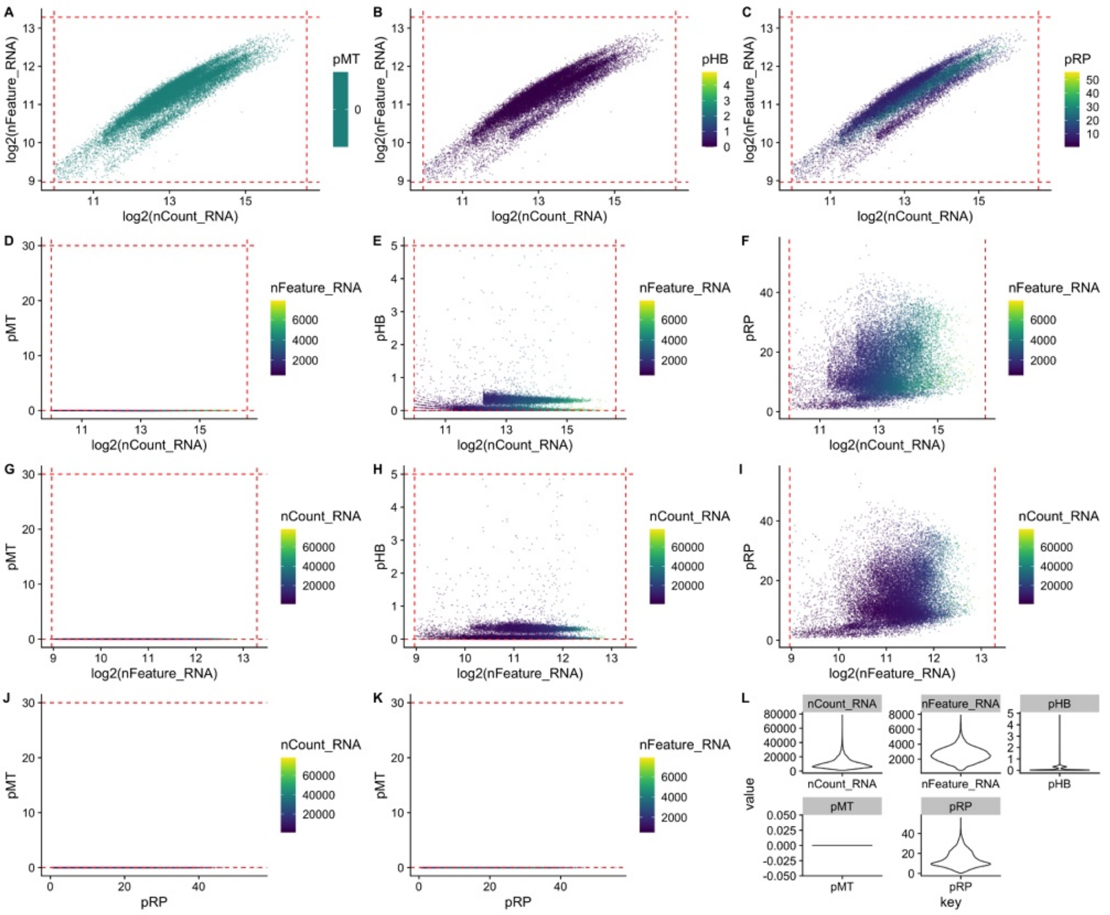

Figure S8. Expression levels of the passage-specific genes in GSE145477. "UMAP" plot was used to visualize the results.

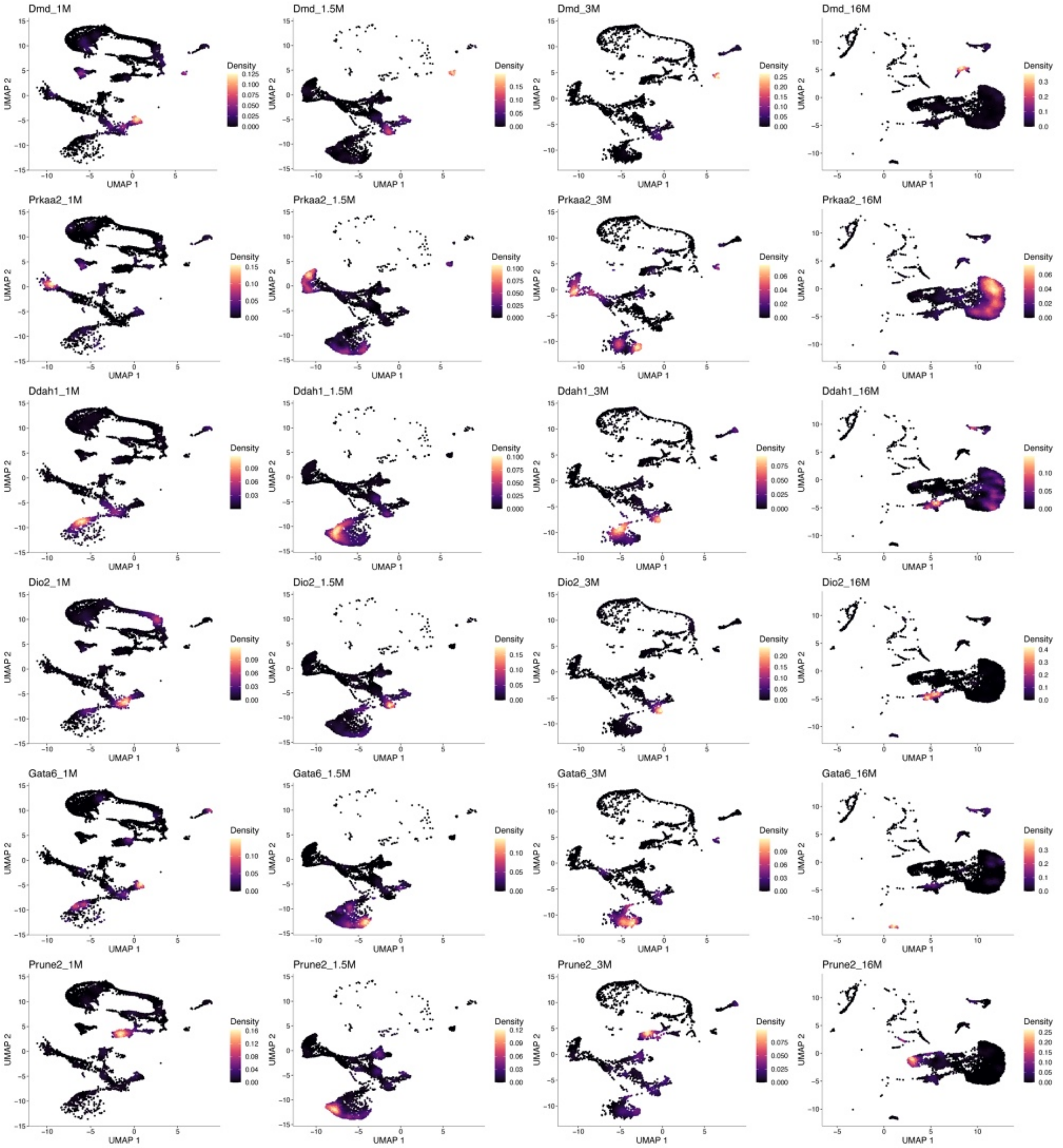

Figure S9. Signature scores of the passage-specific genes in GSE145477. (A) Signature scores of the passage-specific genes in the MSCs from 1-month, 1.5-month, 3-month and 16-month mice. (B) Correlation analysis between passage-specific signature and each passage-specific gene. (C) Violin plot of the signature scores in the MSCs from 1-month, 1.5-month, 3-month and 16-month mice.

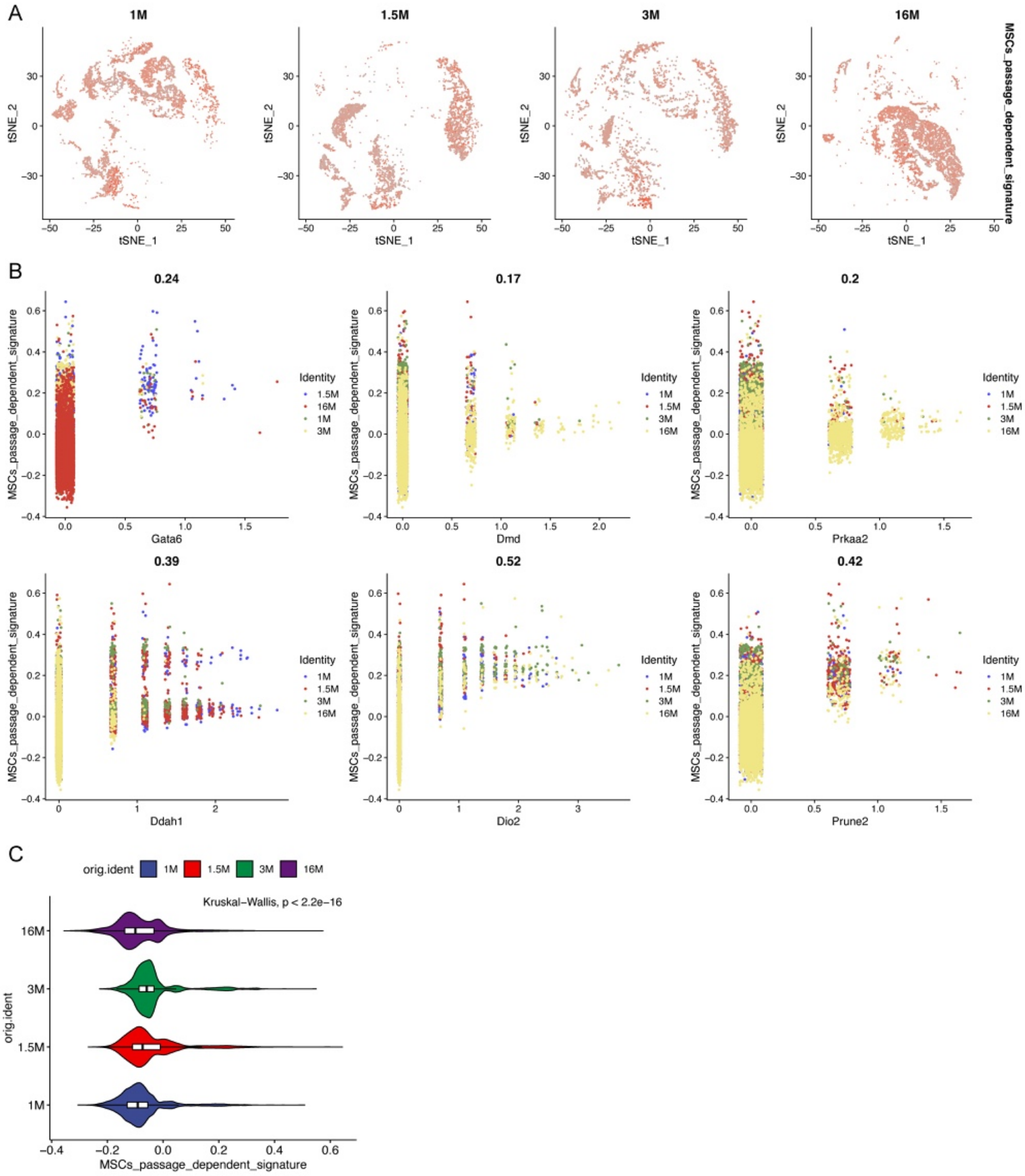

Figure S10. Single cell RNA-seq analysis of GSE117837. (A) Total gene count of single cells derived from different sources. (B) Total read count of single cells derived from different sources. (C) Expression percentage of mitochondria genes in MSCs

derived from different sources after filtering the single cells with stringent criteria. (D) Expression percentage of ribosome protein genes in MSCs derived from different sources after filtering the single cells with stringent criteria. (E) Expression percentage of hemoglobin genes in MSCs derived from different sources after filtering the single cells with stringent criteria. (F) Cell Cycle calculation for MSCs derived from different sources after filtering the single cells with stringent criteria. The "S" phage score is showed. (G) Cell Cycle calculation for MSCs derived from different sources after filtering the single cells with stringent criteria. The "G2M" phage score is showed. (H) Distribution characters of "G1", "G2M" and "S" phase.

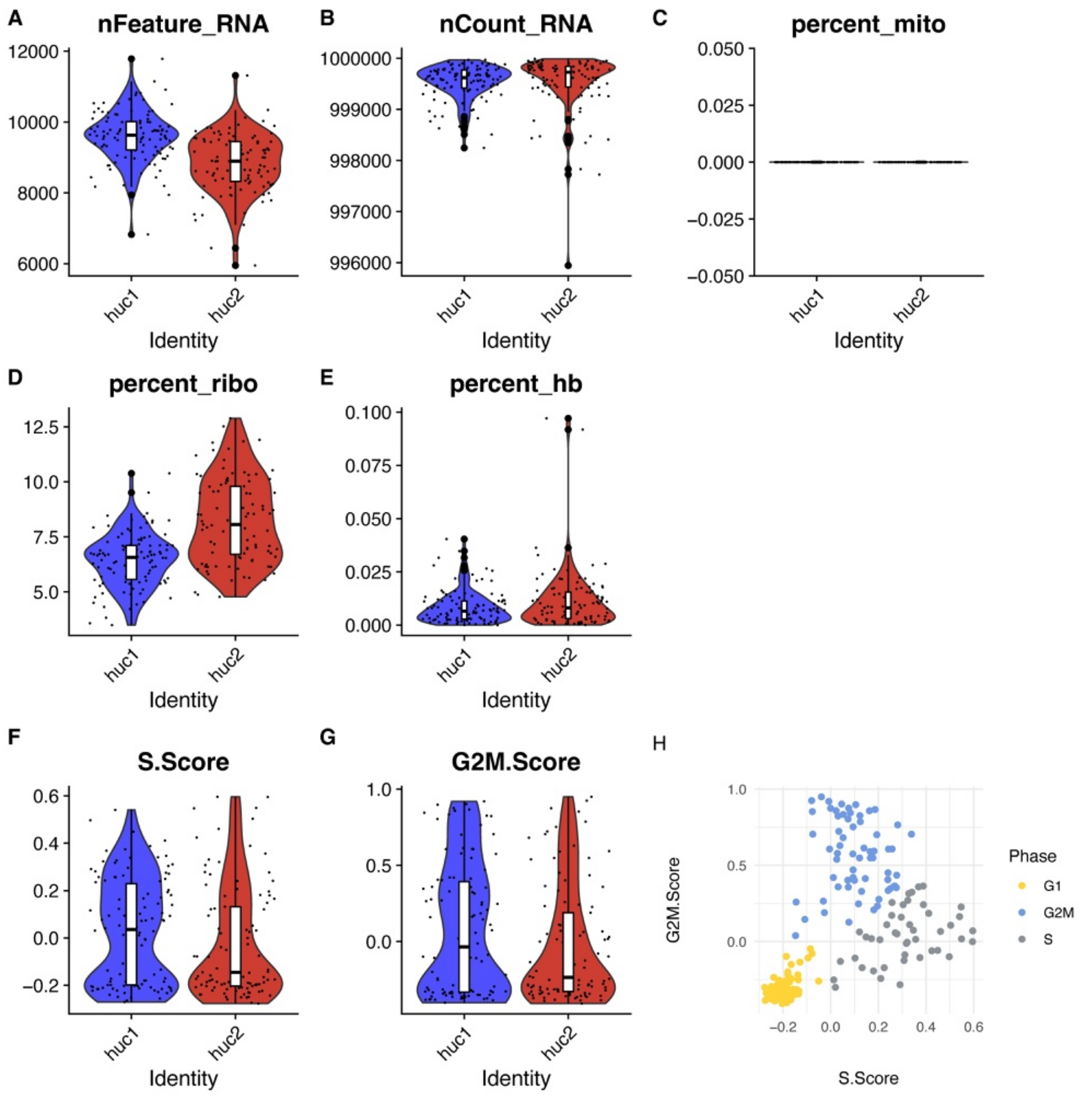

Figure S11. Single cell RNA-seq analysis reveals four MSCs clusters in GSE117837 dataset. (A)(B) Highly variable genes and non-variable genes in GSE117837. (C) PCA analysis of GSE117837. (D) Detailed proportion of MSCs in different clusters and sources. (E) Distribution of clusters in different sources and groups, respectively.

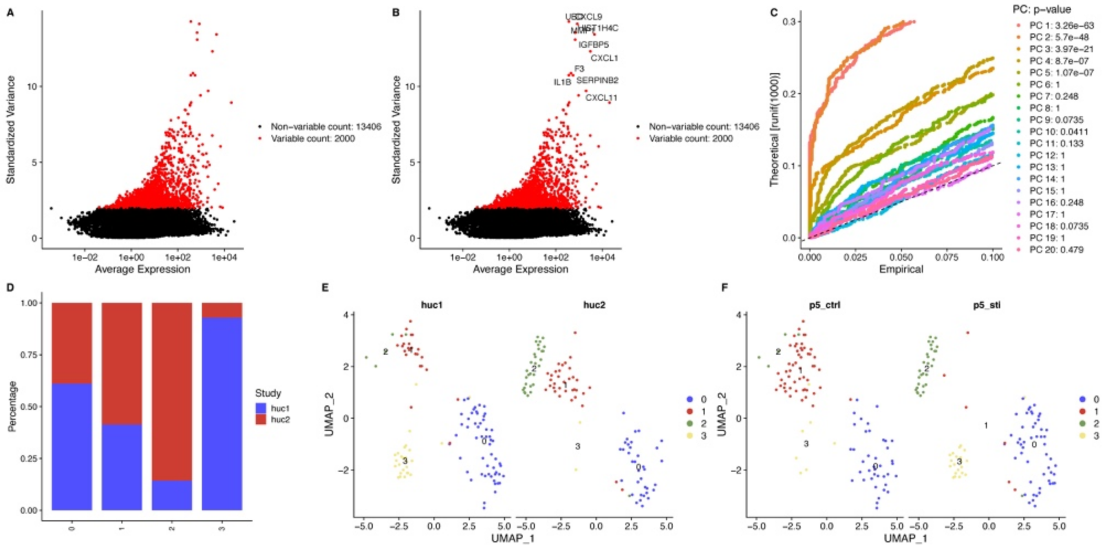

Figure S12. Expression levels of the passage-specific genes in GSE117837. "UMAP" plot was used to visualize the results.

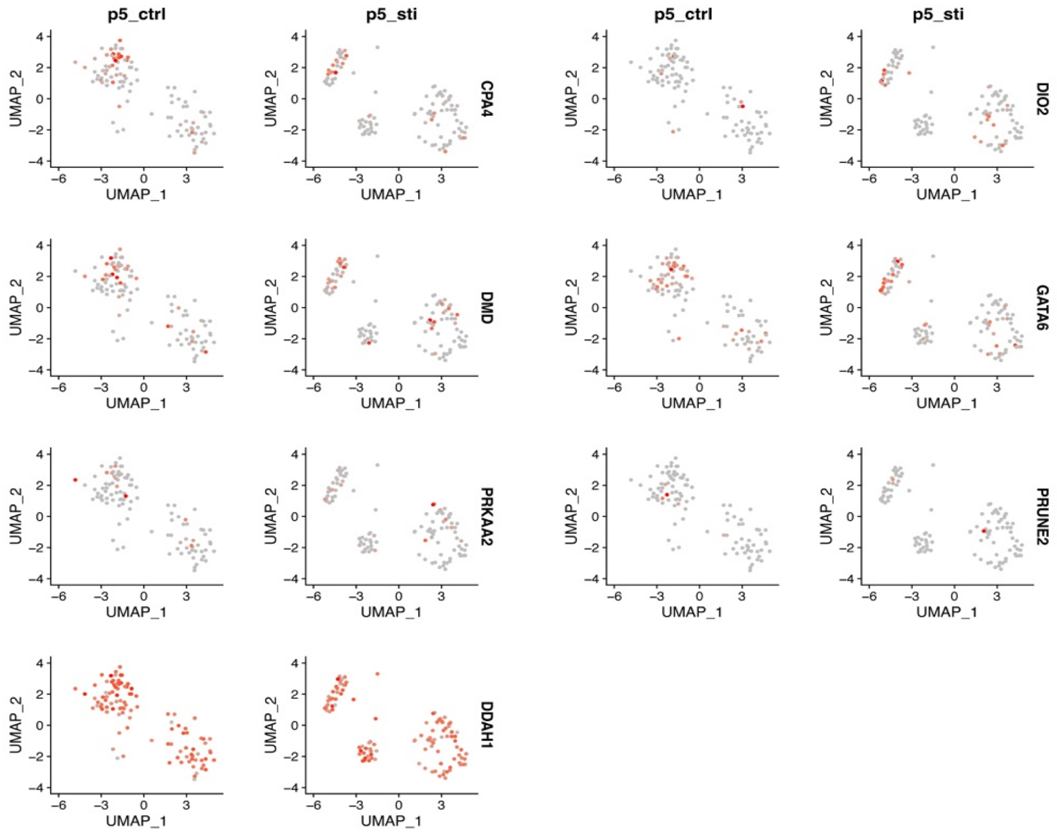

Table S1. Primers of qRT-PCR assay

Name	Accession Number	Sequence (5' to 3')	Tm (°C)
**GAPDH**	NM_001206359.1	ATCCTGGGCTACACTGAGGAC<br>AAGTGGTCGTTGAGGGCAATG	60
**hPRUNE2**	NM_015225.3	CAACGCGCCAAATCTAAACTG<br>GTTCAGCACTGGTAAACACAGA	58
**hCPA4**	NM_016352.4	AGGTGGATACTGTTCATTGGGG<br>TTGCTGATCTCGTCTCCATTTC	60
**hDIO2**	NM_000793.6	TCCTCCTCGATGCCTACAAAC<br>CACAGACTAATTTGCCTTGGGA	59
**hPRKAA2**	NM_006252.4	CTGTAAGCATGGACGGGTTGA<br>AAATCGGCTATCTTGGCATTCA	60
**hDMD**	NM_004006.3	AGCAAGAGCACAACAATTTGGT<br>CCCTGTTCGTCCCGTATCATAA	60
**hDDAH1**	NM_001554.5	CAAAAGGACAAATCAACGAGGTG<br>TGTGCAGATTCACTAGACCCAA	60
**hGATA6**	NM_005257.6	CTCAGTTCCTACGCTTCGCAT<br>GTCGAGGTCAGTGAACAGCA	58

Table S2. The detailed culture media and supplements information of 12 datasets analyzed in the present study.

Dataset	Cell type, Source	Culture media and supplements	Reference
GSE178514	Homo sapiens, Bone marrow	Dulbecco's modified Eagle's medium (DMEM; GIBCO) containing 10% fetal bovine serum (FBS; GIBCO) and supplemented with growth factors (FGF) at 37℃ in a 5% CO_2 atmosphere.	(Wang et al. 2021)
GSE139073	Homo sapiens, Bone marrow	Expansion medium (Dulbecco's Modified Eagle Medium-Low Glucose [DMEM-LG; Sigma-Aldrich] containing 10% fetal calf serum [FCS; Biochrom AG], 100 U/mL penicillin, and 100 μg/mL streptomycin [Biochrom AG], and 2 mM L-alanyl-L-glutamine [GlutaMAX; Gibco]).	(Andrzejewska et al. 2019)
GSE146247	Homo sapiens, Bone marrow	All hMSCs were cultured in MSC culture medium: 90% α-MEM with GlutaMAX (Gibco), 10% FBS (Lot A77E01F, Gemcell), 1% penicillin/streptomycin (Gibco) and 1 ng/mL bFGF (Joint Protein Central). 10 μmol/L Lamivudine (3TC) (L1295- 50mg, Sigma-Aldrich).	(Bi, et al. 2020)
GSE120800	Homo sapiens, Bone marrow	(1) Control MSCs were cultured in low-glucose Dulbecco Modified Eagle Medium (DMEM), 10% fetal bovine serum (FBS; Atlanta Biologicals, Atlanta, GA, USA), and antibiotics.<br>(2) iPSC-MSCs were induced from iPSCs by STEMdiff™-ACF Mesenchymal Induction	(Jiao, et al. 2021)

		Medium for 4 days with daily medium change, then cultured in a complete MesenCult™-ACF Plus Medium for another 2 days with daily medium change. To maintain iPSC-MSCs, the culture medium was then switched to normal growth medium composed of DMEM, 10% FBS, and antibiotics.	
GSE115068	Mus musculus, Adipose	DMEM containing 10% FBS at 37 °C in 5% CO_2.	(Taketani et al. 2019)
GSE145477	Mus musculus, Bone marrow	Cells were cultured in a growth medium (a-MEM supplemented with 15% FBS, 0.1% b-mercaptoethanol, 20 mM glutamine, 100 IU/ ml penicillin, and 100 mg/ml streptomycin) for 7 days before counting CFU-F number.	(Zhong et al. 2020) (Zhong et al. 2022)
GSE25069	Mus musculus, Adipose	Cells were plated at a concentration of 10^5 cells/cm2 in DMEM medium (GIBCO) with high glucose concentration (GLUTAMAX I, GIBCO; 10% FCS, GIBCO; 100 U/ml penicillin; and 100 µg/ml streptomycin). After 2–3 weeks of culture, a homogeneous cell population was obtained.	(Nodari et al. 2021)
GSE183995	Homo sapiens, Umbilical Cord	Human umbilical cord blood-derived mononuclear cells were cultured with a density of 5×10^4 cells/cm^2 in α-minimum essential medium (α-MEM; Gibco. Calsbad, CA, United States) supplemented with 10% (v/v) fetal bovine serum (FBS; Gibco). Cells were maintained at 37°C in a humidified atmosphere with 5% CO2 and Culture	(Kim et al. 2021)

		medium was replaced twice a week.	
GSE137186	Homo sapiens, Bone marrow	Cells were suspended in stem cell expansion medium consisting of low glucose DMEM supplemented with 10 vol%/vol% fetal bovine serum (FBS, Sigma-Aldrich), 1 vol%/vol% Glutamax (Gibco), and 1 vol%/vol% Penicillin/Streptomycin (Sigma-Aldrich). The medium was also supplemented with 10 ng/mL FGF-2 (Peprotech).	(Salerno et al. 2020)
GSE110755	Homo sapiens, Bone marrow	MSCs were expanded by plating at a density of 10,500 cells/T175 flask (60 cells/cm^2) using standard MSC growth medium: 500 ml α-MEM, 6 ml 200 mM l-glutamine, 6 ml 10,000 U/ml penicillin-streptomycin (Life Technologies, Carlsbad, CA, www.lifetechnologies.com), and 100 ml of lot-selected fetal bovine serum (FBS) (JM Bioscience, San Diego, CA, www.jmbioscience.com).	(Lam et al. 2018)
GSE39035	Homo sapiens, Bone marrow	Cells were cultured in minimum essential α-medium (αMEM) supplemented with 20mM HEPES, 10% FBS, 2mM L-glutamine and 100 units/ml penicillin and 100 µg/ml streptomycin (all from Gibco, Invitrogen, Paisley, UK).	(Kilpinen et al. 2013)
GSE117837	Homo sapiens, Umbilical cord	The tissue clumps were cultured in the petri dish with low-glucose DMEM supplemented with 10% FBS, 100 U/mL penicillin, and 100 U/mL streptomycin. Half of culture medium was changed on day 4. At day 12, all the clumps were removed.	(Huang et al. 2019)

Reference

Andrzejewska A, Catar R, Schoon J, Qazi TH, Sass FA, Jacobi D, et al. Multi-Parameter Analysis of Biobanked Human Bone Marrow Stromal Cells Shows Little Influence for Donor Age and Mild Comorbidities on Phenotypic and Functional Properties. Front Immunol. (2019) 10:2474. doi: 10.3389/fimmu.2019.02474

Bi S, Liu Z, Wu Z, Wang Z, Liu X, Wang S, et al. SIRT7 antagonizes human stem cell aging as a heterochromatin stabilizer. Protein Cell. (2020) 11:483-504. doi: 10.1007/s13238-020-00728-4

Huang Y, Li Q, Zhang K, Hu M, Wang Y, Du L, et al. Single cell transcriptomic analysis of human mesenchymal stem cells reveals limited heterogeneity. Cell Death Dis. (2019) 10:368. doi: 10.1038/s41419-019-1583-4

Jiao H, Walczak BE, Lee MS, Lemieux ME, Li WJ. GATA6 regulates aging of human mesenchymal stem/stromal cells. Stem Cells. (2021) 39:62-77. doi: 10.1002/stem.3297

Kilpinen L, Tigistu-Sahle F, Oja S, Greco D, Parmar A, Saavalainen P, et al. Aging bone marrow mesenchymal stromal cells have altered membrane glycerophospholipid composition and functionality. J Lipid Res. (2013) 54:622-635. doi: 10.1194/jlr.M030650

Kim M, Go J, Kwon JH, Jin HJ, Bae YK, Kim EY, et al. CD26 Inhibition Potentiates the Therapeutic Effects of Human Umbilical Cord Blood-Derived Mesenchymal Stem Cells by Delaying Cellular Senescence. Front Cell Dev Biol. (2021) 9:803645. doi: 10.3389/fcell.2021.803645

Lam J, Bellayr IH, Marklein RA, Bauer SR, Puri RK, Sung KE. Functional Profiling of Chondrogenically Induced Multipotent Stromal Cell Aggregates Reveals Transcriptomic and Emergent Morphological Phenotypes Predictive of Differentiation Capacity. Stem Cells Transl Med. (2018) 7:664-675. doi: 10.1002/sctm.18-0065

Nodari A, Scambi I, Peroni D, Calabria E, Benati D, Mannucci S, et al. Interferon regulatory factor 7 impairs cellular metabolism in aging adipose-derived stromal cells. J Cell Sci. (2021) 134. doi: 10.1242/jcs.256230

Salerno A, Brady K, Rikkers M, Li C, Caamano-Gutierrez E, Falciani F, et al. MMP13 and TIMP1 are functional markers for two different potential modes of action by mesenchymal stem/stromal cells when treating osteoarthritis. Stem Cells. (2020) 38:1438-1453. doi: 10.1002/stem.3255

Taketani H, Nishikawa T, Nakajima H, Kodo K, Sugimoto S, Aoi W, et al. Aging-associated impairment in metabolic compensation by subcutaneous adipose tissue promotes diet-induced fatty liver disease in mice. Diabetes Metab Syndr Obes. (2019) 12:1473-1492. doi: 10.2147/DMSO.S214093

Wang S, Wang Z, Su H, Chen F, Ma M, Yu W, et al. Effects of long-term culture on the biological characteristics and RNA profiles of human bone-marrow-derived mesenchymal stem cells. Mol Ther Nucleic Acids. (2021) 26:557-574. doi: 10.1016/j.omtn.2021.08.013

Zhong L, Yao L, Holdreith N, Yu W, Gui T, Miao Z, et al. Transient expansion and myofibroblast conversion of adipogenic lineage precursors mediate bone marrow repair after radiation. JCI Insight. (2022) 7. doi: 10.1172/jci.insight.150323

Zhong L, Yao L, Tower RJ, Wei Y, Miao Z, Park J, et al. Single cell transcriptomics identifies a unique adipose lineage cell population that regulates bone marrow environment. Elife. (2020) 9. doi: 10.7554/eLife.54695
